# The influence of eye level and the horizon on the perception of the size and distance of objects in a 2D scene

**DOI:** 10.1167/jov.26.11.1

**Published:** 2026-11-04

**Authors:** John J.-J. Kim, Laurence R. Harris

**Affiliations:** 1 Department of Psychology, York University, Toronto, ON, Canada; 2 Defence Research and Development Canada, Toronto, ON, Canada

**Keywords:** distance perception, size perception, eye level, horizon, screen size

## Abstract

People can infer the relative distances of objects reliably from their visual size and position in a two-dimensional (2D) scene (e.g., viewed on a computer screen) but not their absolute simulated distance. Might this be an artifact of picture viewing? In a naturally sized indoor corridor scene, projected onto a wall and aligned with the viewer's eye level, participants adjusted the size of the image of an object based on its position in the scene or its position based on its visual size to match the size of an actual object held in their hands (Experiment 1). The adjusted position was consistent with the visual size of the object, but the adjusted size was consistently larger than its veridical size. Such discrepancy demonstrates that the distance and the size of the object in the scene were judged differently, where the visual size of the object relative to its absolute distance may have been judged erroneously. However, expressing the data as the horizon ratio suggested that people might do these tasks using this ratio rather than considering the simulated absolute distance of the object. Measuring people's perceptions of the position of the horizon (Experiment 2) revealed that people are generally accurate at judging the elevation of the horizon in a scene, but people misperceived their eye level when looking at a monitor or when the horizon in the scene was misaligned with their eye level. The misperceived eye level may be due to the misjudged distance between their eyes and the ground (i.e., the eye height) which may lead to errors when computing distance to the object on the ground based on its visual angular declination. Therefore, we concluded that the horizon position and the visually perceived eye level play important role in people's distance and size perception in 2D scenes and the objects in it.

## Introduction

Knowing the distances to various objects in their environment is important for any living creature for survival. Judging the egocentric distance of an object (i.e., its distance from the observer) is a necessary cognitive process to plan actions to either interact with or avoid it, although the distance may not have to be gauged veridically for accurate action toward an object (Durgin, 2014). Most animals cannot recognize objects in static images (e.g., in pictures), at least not without significant training (Jitsumori, 2010). But, humans can easily recognize and perceive objects (e.g., from drawings on a cave wall) and even three-dimensional (3D) space represented in images presented on a flat two-dimensional (2D) surface (e.g., landscape paintings with perspective). As result, people have used images on cave walls, canvas, paper, and more recently display screens that represent 3D spaces for many purposes, from simple entertainment or artistic expression to operating equipment remotely. Studies have shown, however, that the space presented in a 2D scene (e.g., a photograph or a live video shown on a monitor screen) is perceived as smaller than what the image represents—that is, a miniatured world (Kim & Harris, 2022; Roscoe, 1984). We conducted two experiments to investigate the contributing factors to this miniaturization of perceived space when a 3D scene is viewed on a 2D display. We evaluated this phenomenon by measuring people's perception of distance and the size of an object in a 2D scene and how these perceptions may be impacted by the perception of eye level and the elevation of the horizon. We also considered the influence of screen size and of the alignment of the scene with the viewer's eyes.

### Background

When viewing a picture of a scene, two distinct perceptions of space occur simultaneously: (a) the 3D space presented in the picture and (b) the room in which the observer views the picture (Gibson, 1954). In Kim and Harris (2022), we tested whether people can use visual size or position (i.e., distance below the horizon) of an object in a 2D scene to infer its distance. When asked to set the size of a target (representing a smartphone) to match the reference size (a real smartphone held in their hands), some participants made it the full size of the reference as if it were physically present at the distance of the screen rather than at the distance simulated within the scene. This result shows evidence of them failing to perceive the scene on a screen as its own space. However, this study was conducted online with the participants using their own computer monitor in an uncontrolled environment. In another study, Riecke, Behbahani, and Shaw (2009) demonstrated that people can walk, blindfolded, to a position indicated on the ground shown in a photorealistic scene displayed on a screen (aligned with the real world) as accurately as when they saw the target position in the real world. This means that people can infer a real 3D space from a 2D image of a scene, perceiving it accurately when the image is well aligned to a real space. In pictures, binocular cues, such as stereoscopic disparity (Mather & Smith, 2000), are not present and such pictures generally indicate the actual structure of the physical 3D world (e.g., a room surrounding some hanging picture). However, monocular cues, such as visual size (Ittelson, 1951; Kunnapas, 1968) and ground texture (Brenner & Smeets, 2018; Sedgwick, 2021), can be easily depicted in a 2D scene such as a painting or photograph. When binocular cues are not available, the scene may have to be correctly aligned to the viewer's eyes for the person to be able to properly utilize the monocular cues and correctly perceive the spatial properties represented in the image.

#### Alignment of the horizon and the eye level

When looking at a real-world scene, the horizon (when visible) and the observer's eye level are geometrically aligned. However, when a person is looking at a 2D pictorial scene (e.g., projected on a screen or represented in a painting), the horizon (represented with perspective lines and a vanishing point) may or may not correctly align with the eye level of the viewer, as shown in Figure 1.

**Figure 1.**
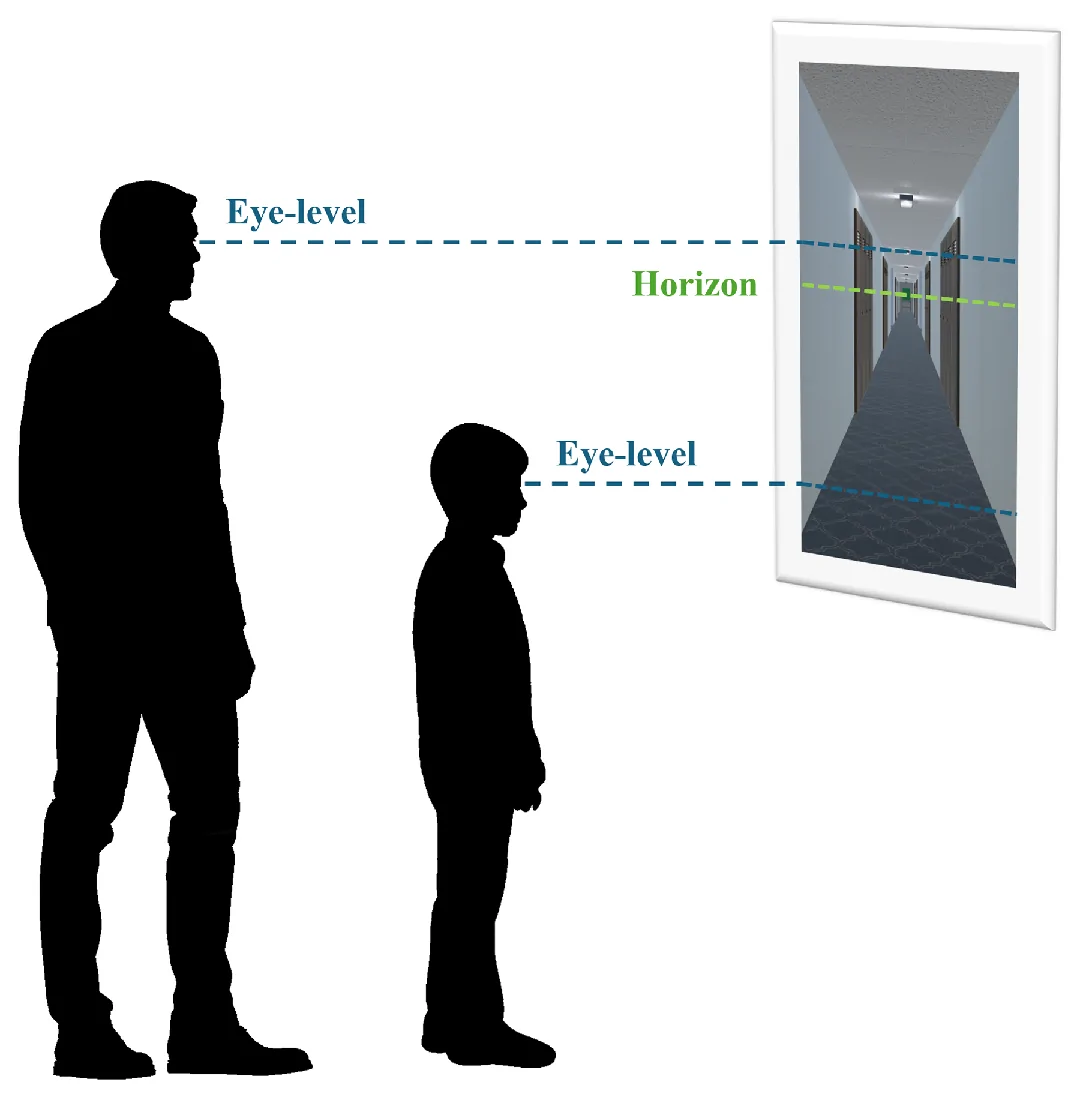
Diagram of the horizon in a visual scene and eye level of the observers. The eye level of each observer could differ with their physical height and posture, and the horizon may or may not have been aligned.

Dixon, Wraga, Proffitt, and Williams (2000) noted such a disconnect and argued that, for people to effectively see the image at the correct scale, not only does the horizon have to be correctly aligned with their eye level but observers also have to actively perceive it as such. In contrast, Rogers (1996) suggested that people might just assume that the horizon is aligned with their eye level when the horizon is not visible. These are conflicting points of view. Neither of these studies, and no other studies that we know of, directly measured people's perceived elevation of the horizon in a pictorial scene or their eye level. Therefore, the direction of such influence between them is unclear and should be evaluated further.

Furthermore, knowing one's eye height (i.e., the distance between one's eye level and the ground) is important for computing the angular declination of an object on the ground (Ooi, Wu, & He, 2001). Misperceiving eye level can lead to misjudging eye height and can significantly influence the estimate of the distance to objects in the room and even of the dimensions of the room (Leyrer, Linkenauger, Bülthoff, Kloos, & Mohler, 2011) and of the height of surfaces in the room (Mark, 1987). Such an effect may contribute to a scene being perceived as miniaturized when presented on a 2D display (Kim & Harris, 2022; Roscoe, 1984).

#### Size versus distance perception

Gauging egocentric distance uses various visual cues even in pictures. Providing more distance information such as other objects in the visual scene have been shown to improve people's accuracy in perceiving both the distance and size of an object (Brenner & van Damme, 1999). However, some studies show evidence that distance information in a real-world scene, when displayed on a screen, affects judgment of size but not distance (Meehan & Triggs, 1992). For example, the presence of familiar objects in a scene has been shown to improve size judgment but not distance judgment (Kim & Harris, 2022). The familiar size effect—the influence of prior knowledge of the typical size of an object when judging its size and distance—is also stronger when viewing an object monocularly compared to viewing it binocularly for perceived size, but not for perceived distance (Maltz, Stubbs, Quinlan, Rzepka, Martin, & Culham, 2021). Thus, various monocular distance cues added in a 2D scene may impact participants’ size and distance perceptions differently.

### Hypotheses

In Experiment 1, we evaluated people's ability to judge the distance and size of an object within an accurately scaled realistic 2D scene representing a 3D space, presented with rich distance information. The horizon in the scene was aligned with the viewer's eye level, and the experiment was conducted both monocularly and binocularly. We expected that people would be accurate at the distance and size judgment tasks as demonstrated with a photorealistic scene aligned with a real room (Riecke et al., 2009). However, in our previous study (Kim & Harris, 2022), the presence of familiar objects in the scene only helped when people matched object position to the reference based on its visual size and not when matching size based on target position. Therefore, we hypothesized there would be a similar task-dependent effect from the monocular distance cues we added in the present study, enhancing people's size perception more than their distance perception. We also hypothesized that people would be more accurate at judging object size when viewing the scene monocularly because of the more accurately perceived size in the absence of conflicting binocular cues. We expected no difference between the viewing conditions when judging distance, as past evidence showed that monocular distance information may affect size perception but not distance perception (Maltz et al., 2021; Meehan & Triggs, 1992).

In Experiment 2, we measured people's perceived elevation of the horizon in the scene (i.e., the horizon position) and their visually perceived eye level using a scene projected onto a wall, identical to Experiment 1. The same hallway scene was also presented on a conventional computer monitor. The horizon position and the eye level of the observers were misaligned in some conditions to assess their relationship. We hypothesized that people would make larger errors on a computer monitor than on a projection on a wall, as suggested from our previous study results (Kim & Harris, 2022). We also hypothesized that people would assume the horizon was aligned with their eye level as Rogers (1996) suggested, misjudging the horizon position when it was not aligned with their eye level.

## Experiment 1

In Experiment 1, participants adjusted the size or position of a target presented in a 2D hallway scene to match it with a reference size at various simulated distances. The experiment was 2 × 2 mixed design, with *task* as the between-subject factor and *viewing condition* as the within-subject factor. The tasks were either a Position-to-Size task, where participants matched the size of a target in a 2D scene to a reference size by adjusting its size, or a Size-to-Position task, in which they adjusted its position on the screen. These experimental tasks were identical to those of Kim and Harris (2022), but the target and the 2D scene were presented on a large projection on a wall. The viewing conditions were monocular (looking at the scene with their right eye regardless of which eye was dominant) and binocular (looking with both eyes). Our hypotheses were that (a) there would be larger errors in the Position-to-Size task than in the Size-to-Position task, and (b) there would be smaller errors for the monocular viewing condition than for the binocular viewing condition in the Size-to-Position task.

### Methods for Experiment 1

#### Participants

Forty undergraduate students participated in the experiment in the period between November 21, 2023, and March 12, 2024. One participant was removed for an incomplete dataset after the data cleaning process, leaving 39 participants (19 females and 20 males; average age, 19.9 ± 3.08 years) for the final analysis. All participants were students recruited via the Undergraduate Research Participant Pool (URPP) from York University, Toronto, ON, Canada. They had normal or corrected-to-normal vision and signed an informed consent form before participating. Students were given course credit for their participation. The experiments were approved by York University's Ethics Review Board and conducted in accordance with the relevant guidelines and regulations. It is important to note that the participants were from a demographic living in an urban environment familiar with digital devices. The findings from the present study may not generalize in the population not familiar with digitized displays or photographs.

#### Apparatus

The experiment was programmed using PsychoPy 2024.2.4 with Python, which ran on a Windows 10 computer (Alienware 17 R4; Dell Technologies, Round Rock, TX) with an Intel Core i7-7700HQ CPU (2.80 GHz; Intel, Santa Clara, CA), 16.0 gigabyte RAM, and a GeForce GTX 1070 graphics card (NVIDIA, Santa Clara, CA). The scene was made using Unity3D, then a screenshot was taken looking into the hallway to create a 2D image of the scene (with a camera view 1.2 meters above the ground), which was projected on a wall using a short-throw BenQ digital projector (HT1085ST; full HD, 1080p; BenQ, Taipei, Taiwan) (see Figure 2a). Participants used a hand-held mouse (Figure 2b) to adjust the size of the target or its position in the scene.

**Figure 2.**
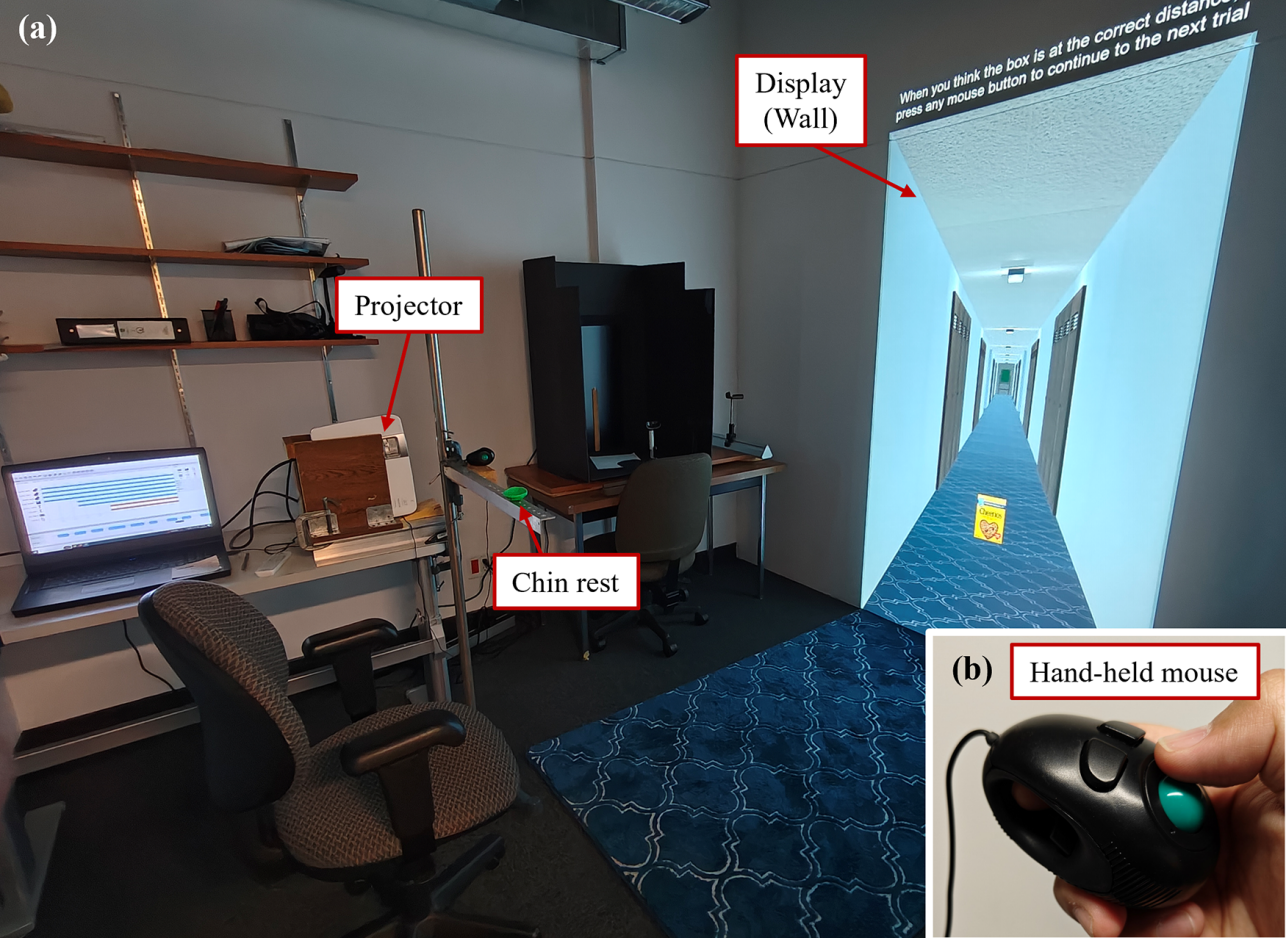
Apparatus and projection setup. (**a**) Test room setup with a chair, chin rest, and projector presenting visual stimuli on the wall. Note that the carpet in the room was continuous with the same-patterned carpet in the image. (**b**) Hand-held mouse that the participants used to adjust target size/position.

#### Experimental setup and stimuli

Participants sat on a chair and looked at the scene with their chin on a chin rest (1.08 meters above the floor) (Figure 2a) to maintain a constant eye level. The height of the chin rest above the floor (of the real and simulated room) was not adjusted for individual subjects, and we approximated the average distance from chin to pupils as 0.12 meter. The eye height and the elevation of the horizon in the simulated scene were aligned at 1.20 meters from the ground. Participants were instructed to imagine they were sitting on a chair looking into a real hallway. To give participants the perception of looking into a real hallway, a correctly scaled 2D scene of an indoor hallway (2.205 meters high and 1.245 meters wide with a simulated length of 25 meters) (Figure 3a) was projected on a wall (2 meters away from the participant's eyes). A real carpet was also laid on the ground from the observer's feet to the wall, lined up with the scene floor and with a matching pattern.

**Figure 3.**
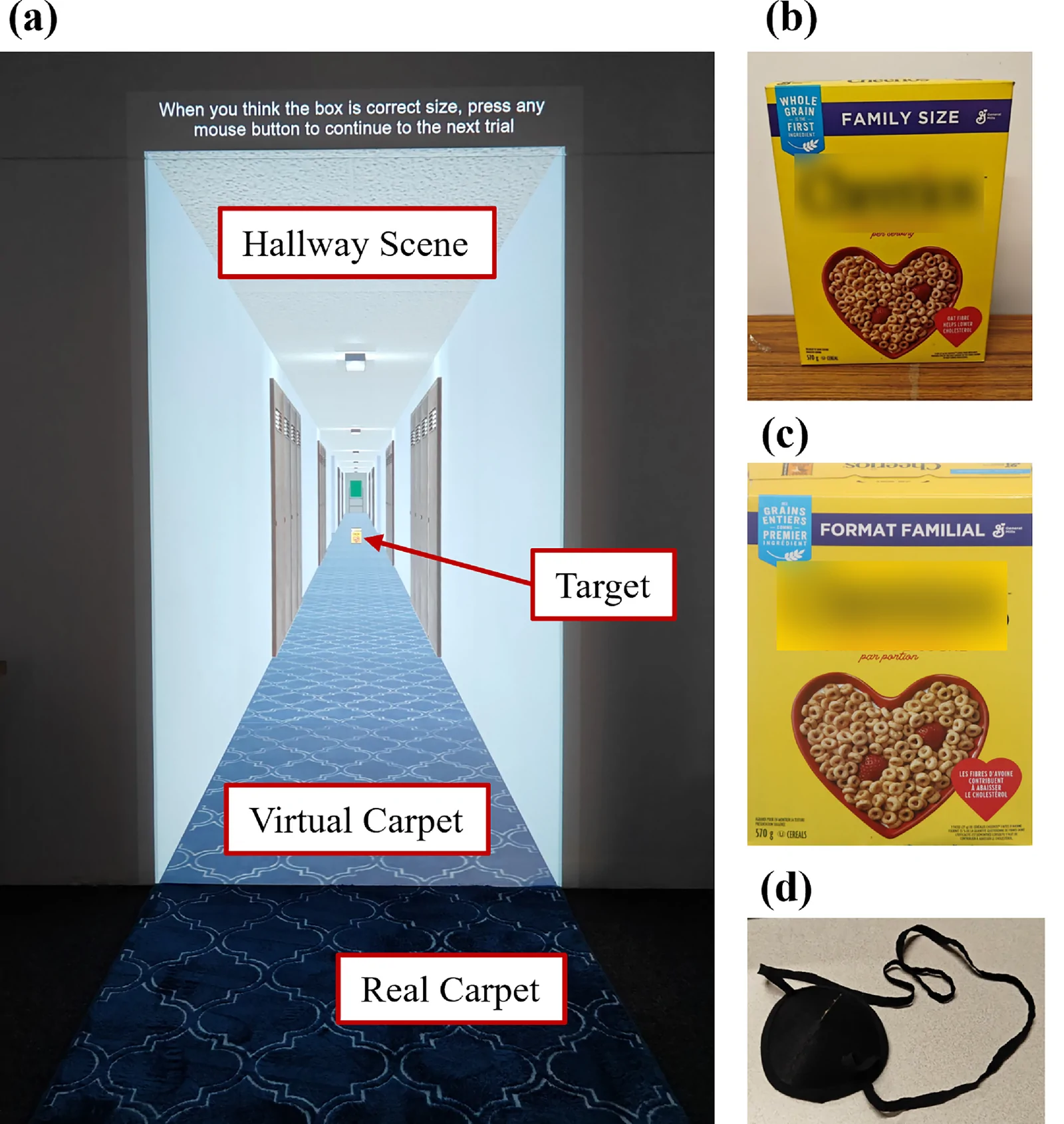
(**a**) The hallway scene, projected on a wall, containing lockers on the side walls, ceiling lights, a green board, and virtual carpet. There was a real carpet with identical color and pattern on the ground in front of the projected scene positioned so it looked as though it continued into the hallway floor. (**b**) The physical reference box, a family-size cereal box, held by participants during the experiment. (**c**) The target image, a photograph of the physical reference box. (**d**) The eye patch used for the monocular viewing conditions.

The target presented in the scene was a photograph of a cereal box (Figure 3c) standing on the ground. The photograph was taken 2 meters away at a lens height of 1.2 meters. Participants held a family-sized cereal box (0.344 meter in height, 0.241 meter in width, and 0.076 meters thick) (Figure 3b) during the experiment as a physical reference size representing the target. The size and position of the target on the screen varied for each simulated distance. Figure 4 illustrates the relationship used to determine them for some example distances. The target size on the screen was represented as scale factors (*S_scale_*) between the stimulus size (*S_stimulus_*) and the simulated size (*S_simulated_*), equivalent to the ratio between the stimulus distance (*D_stimulus_*) and the simulated distance (*D_simulated_*), as shown in Equation 1. The target position on the screen (*P_base_*) was the distance of the base of the target below the horizon.

**Figure 4.**
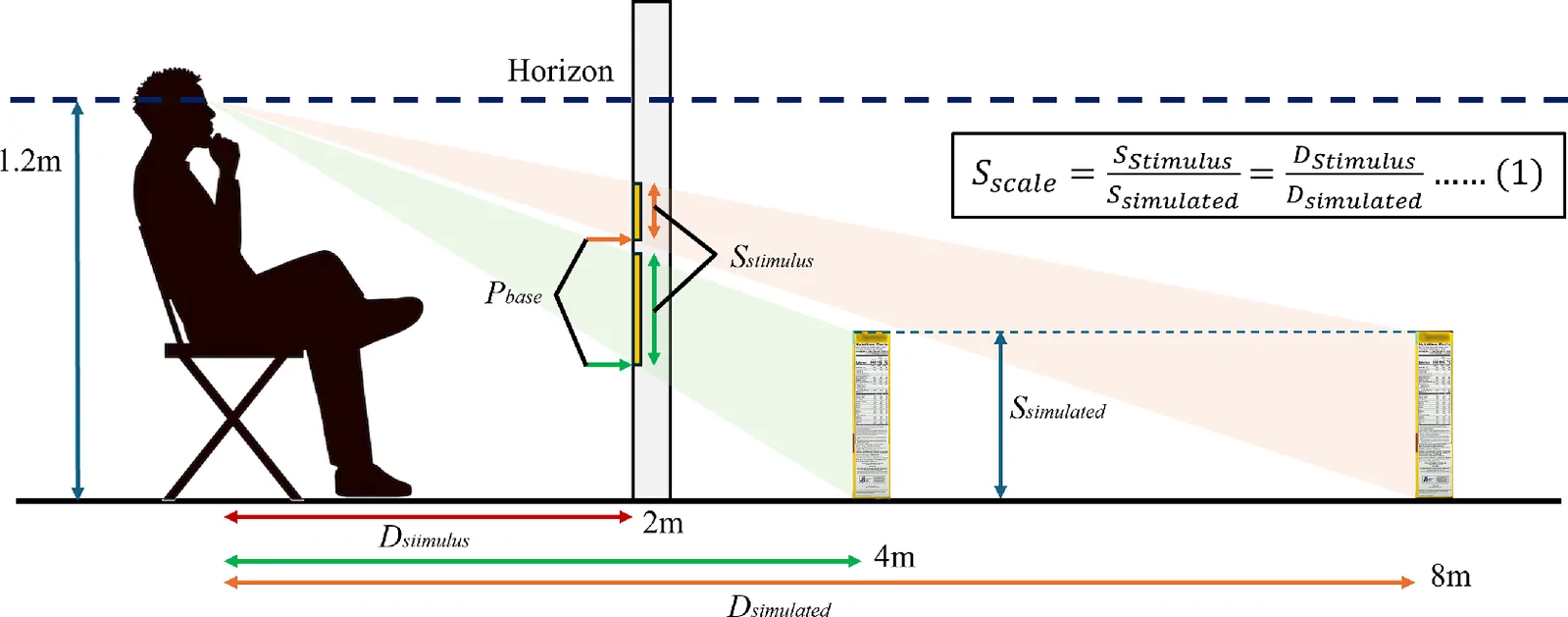
An example of the relationship between the size of the target and its base position in relation to the simulated distance presented on the screen graphically. *S_stimulus_* is the target size presented on the screen, *S_simulated_* is the real size of the target (i.e., the reference box size), *D_stimulus_* is the distance to the target stimulus (i.e., the screen), *D_simulated_* is the simulated target distance, and *P_base_* is the base of the distance of the target below the horizon on the screen.

The target size and position presented on the display were computed using the following formula:

(2) $$\begin{array}{c} P_{base}=\left(m\times \;S_{scale}\right)+b\; \end{array}$$

where *S_scale_* was between 0 (target not visible) and 1 (full size of the simulated box). *P_base_* was between 0.015 (the position of the horizon on the screen, and also *b*) and −0.515 (the position of the bottom of the screen below the horizon, and also *m*). We assumed a linear relationship between *S_scale_* and *P_base_*. Refer to Appendix A for details on the computations and justification of Equation 2.

#### Tasks

During the Size-to-Position and Position-to-Size tasks, participants compared the target with the reference box they held in their hands and adjusted the target to match it to the reference box size. In the Size-to-Position task, they viewed a target presented in the hallway scene sized (*S_stimulus_*) to simulate experimental distances (*D_simulated_*). They then adjusted its position up or down until the target appeared to be the same size as the reference box at the simulated distance for its visual size. The final adjusted positions of the target on the screen (*P_base_*) were recorded.

In the Position-to-Size task, they viewed a target presented in the hallway scene positioned (*P_base_*) to simulate experimental distances (*D_simulated_*). They then adjusted its size until the target appeared to be the same size as the reference box standing at the simulated distance. The final adjusted sizes of the target on the screen (*S_scale_*) were recorded.

#### Task order

Participants did the tasks in one of the two randomly assigned task orders: Size-to-Position then Position-to-Size (SPS) or Position-to-Size then Size-to-Position (PSP). Regardless of the task order, participants’ responses from the first task were used to determine the target size and position for the second task. For example, when the Position-to-Size task was done first, the response sizes to which the participant set the targets were saved, averaged, and used in the subsequent Size-to-Position task. Therefore, participants first determined the size at the geometrically correct (i.e., veridical) target positions and then determined the position from those response sizes (PSP). If people could reliably compute the target size from its position and vice versa, then the final position in the second task should be the same as the initial position regardless of the size they had set the target to in the first task. This process was reversed for the other task order (SPS).

#### Procedure

Participants sat on a chair in the test room (shown in Figure 2a) with their chin on the chin rest and looked at the wall on which the visual stimuli were projected. They then held a mouse (Figure 2b) in their right hand with their fingers and the reference box (Figure 3b) using both hands with their palms on the opposite diagonal corners. They viewed the stimuli either monocularly (wearing an eye patch on their left eye) (Figure 3d for the photograph of the eye patch) or binocularly (no eye patch). Half of the participants started with the binocular condition followed by the monocular condition; the other half did the experiment in the opposite order. The order of the viewing conditions was counterbalanced within each task order group.

The experiment contained five simulated target distances (4, 8, 12, 16, and 20 meters), which were simulated using either the visual size of the target (for Size-to-Position task) or the position of the target (for Position-to-Size task) in the scene projected on the wall. Refer to Figure 4 and Equation 2 for the computations. The order of the target distances presented within each task block was randomized.

For each trial, participants fixated on a fixation cross (no scene) for 1 second, then the target was presented in the test scene. Participants adjusted either the target position or its size depending on the task. They were instructed to make the target appear to be the same size as the reference box “as accurately as possible” at the distance it appeared to be. After participants adjusted the target to the position (Size-to-Position task) or the size (Position-to-Size task) that they thought was correct for its visual size or position, they pressed a mouse button to record their response (*P_base_* or *S_scale_*, respectively) and proceeded to the next trial. The experiment was comprised of 30 trials (5 target distances × 6 trials) in each block, with a total of four blocks (2 viewing conditions × 2 tasks). The sequences are summarized in Figure 5.

**Figure 5.**
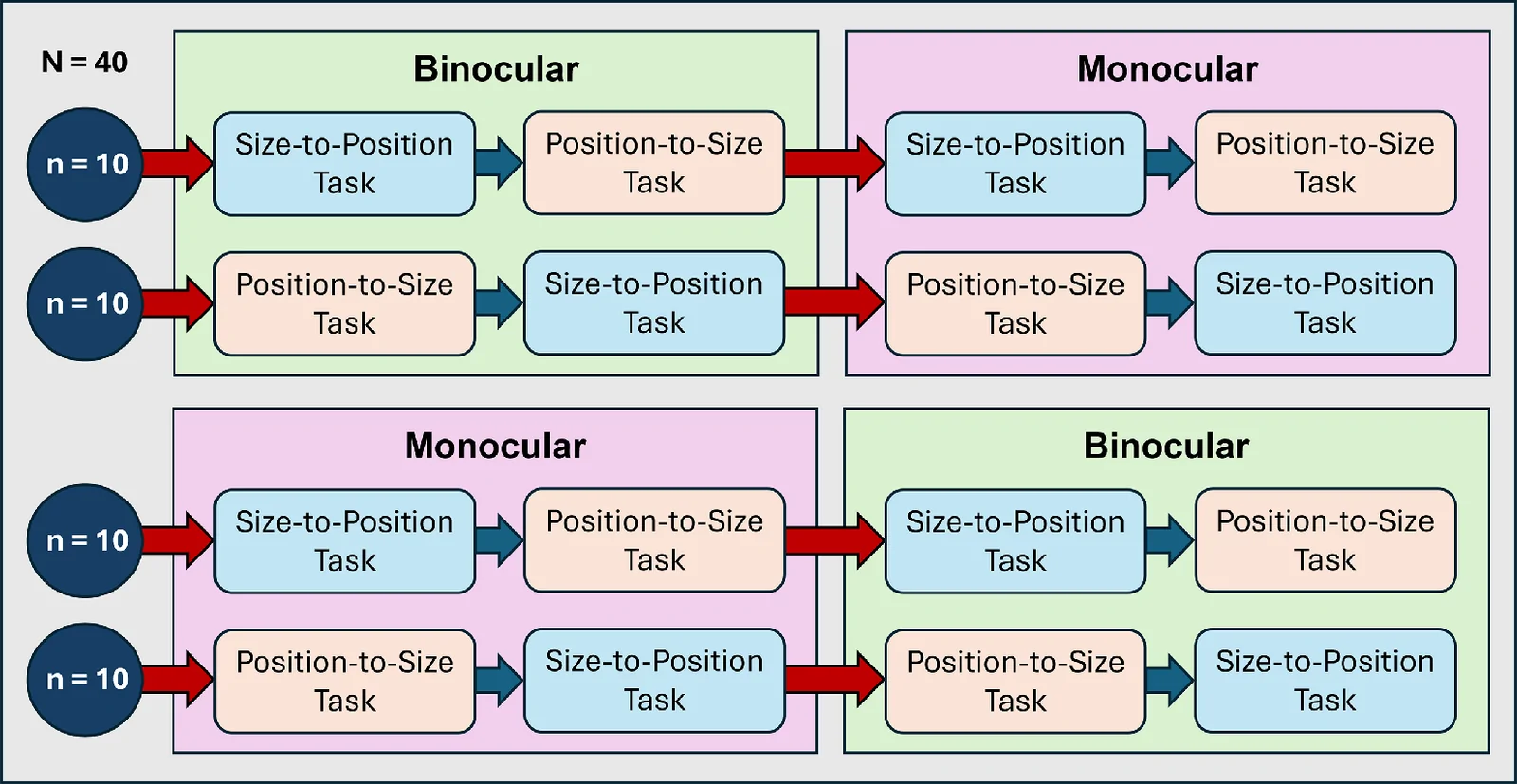
Experimental conditions. Out of the 40 participants, 20 did the binocular condition first followed by the monocular condition, and the other 20 did the opposite. Within each viewing condition order, 10 did the Size-to-Position task first and then the Position-to-Size task (SPS group) in each viewing condition; the other 10 did the opposite order (PSP group).

At the end of each viewing condition block, participants filled in a questionnaire (NASA Task Load Index [TLX]) (Hart & Staveland, 1988) which asked about the mental workload they required to do the task (see Appendix B for the questions) to evaluate whether participants considered it to be more difficult to perform the tasks monocularly or binocularly. They were also asked to write down any strategies they used in an open answer format. The entire experiment lasted approximately 1 hour.

#### Data analysis

A family-wise error rate of 0.05 was used for all analyses, controlled with the Holm–Bonferroni method for adjusting *p* values in multiple comparisons for each analysis of variance (ANOVA) conducted. For any ANOVA violating the assumption of sphericity, the degrees of freedom were adjusted using the Huynh–Feldt correction as the estimated epsilon (ε) was generally less than 0.75.

##### Data cleaning process.

Each participant's responses—position (*P_base_*) from the Size-to-Position task and size (*S_scale_*) from the Position-to-Size task—were evaluated for any possible misunderstanding of the tasks and irrational responses (i.e., target positioned above the horizon, *P_base_* > 0.015). We considered any such responses as errors and removed them from the dataset prior to the analysis. This process removed 98 data points from the total dataset (2.04%). One participant had to be removed from the analysis due to having an incomplete dataset after this process.

##### Analyzing the participant responses.

Two separate mixed-design ANOVAs were conducted to analyze the average responses from each task. We compared *P_base_* (from Size-to-Position task) and *S_sc__ale_* (from Position-to-Size task) across simulated target distances (4, 8, 12, 16, and 20 meters), between task orders (SPS and PSP), and viewing conditions (monocular or binocular). The participants’ responses from each task (*S_scale_* and *P_base_*) were then compared against the veridical size and position for each simulated target distance to evaluate their accuracy.

##### Analyzing the differences between tasks.

The data from the two tasks were from different paradigms: positions (*P_base_*) in the Size-to-Position task and sizes (*S_scale_*) in the Position-to-Size task. Therefore, participant responses from the two tasks had to be standardized. We employed two models to compute the perceived relationship between target size and position in a 2D scene.

For the linear model, the stimuli size and position presented in the experiment were determined with Equation 2, which assumed a linear relationship between them. To verify whether participant responses corresponded with this assumption, the correlation coefficients (*r*) between the stimuli positions and sizes and the participant responses in each task were computed. When the linear relationship was verified, then we fit them with a linear model to estimate the intercept (*b*) and slope (*m*). For the Size-to-Position task, the model was fit with stimulus sizes and response positions. For the Position-to-Size task, the model was fit with stimulus positions and response sizes. These estimated intercepts and slopes were compared between tasks and against the veridical values (0.015 for *b* and 0.515 for *m*).

For the horizon-ratio model, the ratio between the size of the object and the distance to its base from the horizon in a 2D scene was computed for each task. The ratios were compared between tasks and simulated distances. We also compared them against the correct ratio for our test setup (0.287; computed by 0.344 m/1.2 m).

### Results of Experiment 1

#### Task responses compared between conditions

A mixed-design ANOVA revealed significant main effect of distance for both the Size-to-Position task, *F*(1*.*244, 46*.*016) = 592.831, *p* < 0.001, $\eta_{p}^{2}$ = 0.942, and the Position-to-Size task, *F*(1*.*263, 47*.*713) = 534.185, *p* < 0.001, $\eta_{p}^{2}$ = 0.935, verifying that the participant responded to different distances (simulated with size and position of the target) as intended. Post hoc comparisons between distances confirmed this where targets with smaller *S_scale_* (simulated to be farther away) were placed at higher *P_base_* (closer to horizon) or larger *S_scale_* at lower *P_base_* in the Size-to-Position task. In the Position-to-Size task, targets at higher *P_base_* (simulated to be farther away) were made smaller in *S_scale_* or targets at lower *P_base_* were made larger in *S_scale_*.

The ANOVAs showed no significant effect of task order in either the Size-to-Position task, *F*(1, 37) = 2.074, *p* = 0.158, $\eta_{p}^{2}$ = 0.053, or the Position-to-Size task, *F*(1, 37) = 0.75, *p* = 0.678, $\eta_{p}^{2}$ = 0.005. There was also no significant effect of viewing condition in the Size-to-Position task, *F*(1, 37) = 0.249, *p* = 0.621, $\eta_{p}^{2}$ = 0.007, and the Position-to-Size task, *F*(1, 37) = 0.932, *p* = 0.341, $\eta_{p}^{2}$ = 0.025. No interactions between any of the conditions were found in either task. See Tables C1 and C2 in Appendix C for the detailed results of the Size-to-Position and Position-to-Size tasks.

**Table C1.** Mixed-design ANOVA results for Experiment 1, comparing participant responses between all conditions. *p* < 0.05 is indicated in bold. *SS*, sum of squares; *df*, degrees of freedom.

Variable	*SS*	*df*	*Mean* *square*	*F*	*p*	$\eta_{p}^{2}$
(a) Size-to-Position task						
Within-subject effects						
Viewing condition	2.96 × 10^−4^	1	2.96 × 10^−4^	0.249	0.621	0.007
Viewing condition × task order	6.59 × 10^−5^	1	6.59 × 10^−5^	0.055	.815	0.001
Error	0.044	37	—	—	—	—
Distance	1.900	1.244	1.528	592.83	**<0.001**	0.941
Distance × task order	0.004	1.244	0.003	1.350	0.259	0.035
Error	0.119	46.016	0.003	—	—	—
Viewing condition × distance	0.004	1.703	0.002	3.080	0.061	0.077
Viewing condition × distance × task order	0.001	1.703	7.59 × 10^−4^	1.060	0.343	0.028
Error	0.045	63.021	7.17 × 10^−4^	—	—	—
Between-subject effects						
Task order	0.011	1	0.011	2.074	0.158	0.053
Error	0.203	37	0.005	—	—	—
(b) Position-to-Size task						
Within-subject effects						
Viewing condition	0.005	1	0.005	0.932	0.341	0.025
Viewing condition × task order	0.002	1	0.002	0.327	0.571	0.009
Error	0.213	37	0.006	—	—	—
Distance	8.032	1.263	6.362	534.19	**<0.001**	0.935
Distance × task order	0.018	1.263	0.014	1.211	0.289	0.032
Error	0.556	46.713	0.012	—	—	—
Viewing condition × distance	0.004	2.068	0.002	1.141	0.326	0.030
Viewing condition × distance × task order	0.003	2.068	0.002	0.904	0.412	0.024
Error	0.137	76.513	0.002	—	—	—
Between subject effects						
Task order	0.012	1	0.012	0.175	0.678	0.005
Error	2.516	37	0.068	—	—	—

**Table C2.** Post hoc comparisons results for Experiment 1, comparing participant responses between distances.

Distances (m)		*M_diff_*	*SE*	*df*	*t*	Cohen's *d*	*p_holm_*
(a) Size-to-Position task							
4	8	−0.121	0.005	37	−23.365	−3.630	<0.001
8	12	−0.039	0.002	37	−16.200	−1.169	<0.001
12	16	−0.021	0.001	37	−15.404	−0.635	<0.001
16	20	−0.011	0.002	37	−6.689	−0.341	<0.001
(b) Position-to-Size task							
4	8	0.241	0.011	37	21.217	2.510	<0.001
8	12	0.082	0.004	37	18.949	0.854	<0.001
12	16	0.047	0.004	37	13.497	0.493	<0.001
16	20	0.027	0.003	37	9.350	0.280	<0.001

##### Self-reported mental load between viewing conditions.

Although participant responses did not significantly differ between viewing conditions, the NASA TLX scores (self-reported after each viewing condition) compared using an independent *t*-test showed that doing the task monocularly (*M* = 49, *SD* = 14) required significantly more mental load compared to doing it binocularly, *M* = 40, *SD* = 10, *t*(39) = 5.00, *p* < 0.001, Cohen's *d* = 0.79. According to the score interpretation suggested by Prabaswari, Basumerda, and Utomo (2019) with equal weight for each question, doing the task required a “somewhat high” mental workload (scores between 30 and 49) in both viewing conditions on average.

#### Comparing participant responses to the veridical positions and sizes

Because there was no statistically significant effect of task order or viewing condition, we averaged the responses between the viewing conditions and combined the responses together between the two task order groups. The deviations in the average responses, *P_base_* (from Size-to-Position task) and *S_sc__ale_* (from Position-to-Size task), for each simulated target distance (4, 8, 12, 16, and 20 meters) from veridical were tested using one-sample *t*-tests with corrected *p* values using the Holm–Bonferroni method. The response data are plotted in Figures 6a and 6b.

**Figure 6.**
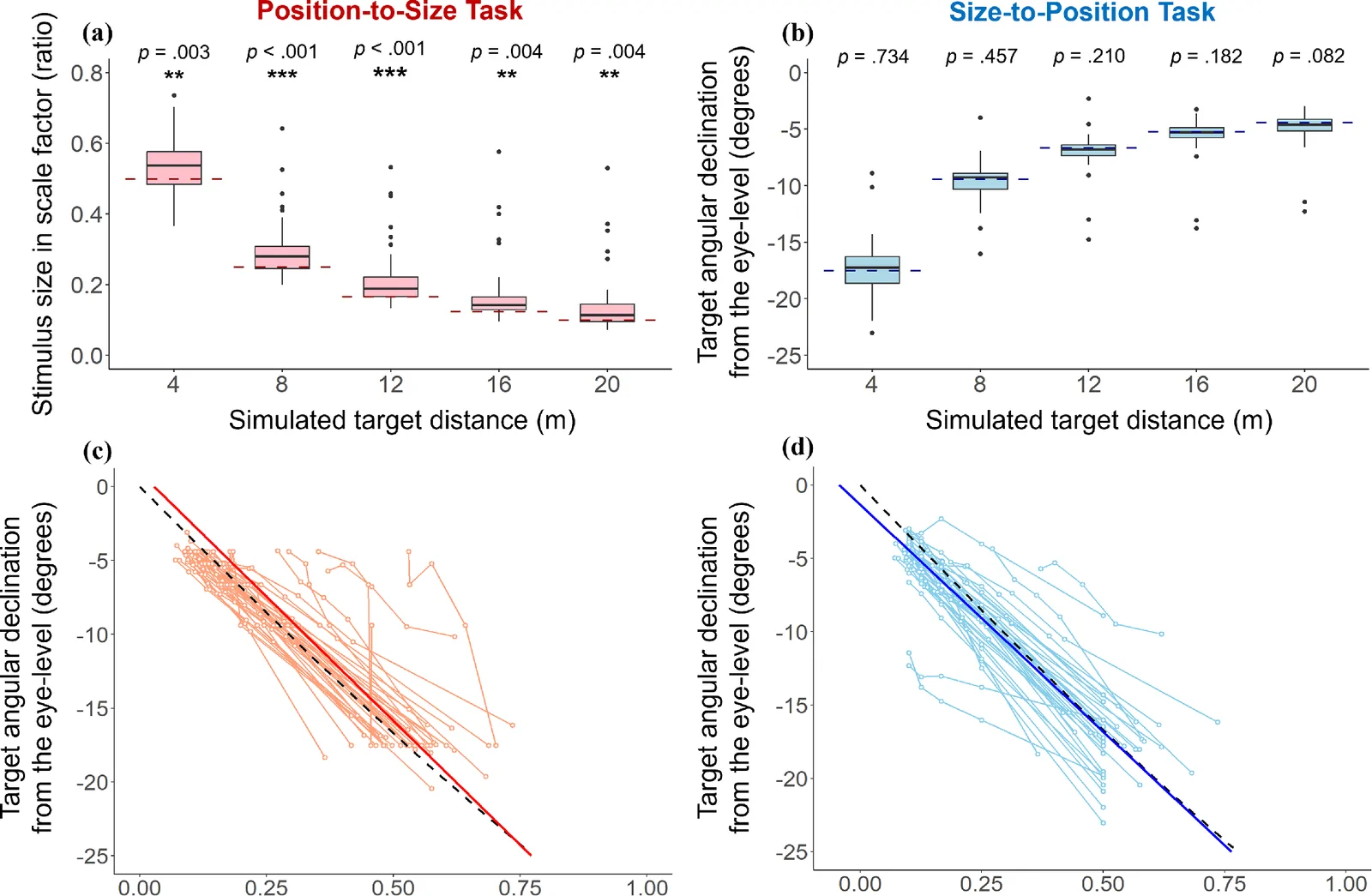
Target size and position for each task in Experiment 1. (**a**, **b**) The box plots (with medians) show the response sizes, where the *y*-axis represents the angular declination of the target (**a**) and the response positions, where the *y*-axis represents the stimulus size in scale factor compared with the veridical values for each simulated target distance (i.e., the dashed lines) (b). *p < 0.05, **p < 0.01, *** *p* < 0.001. (**c**) The relationship between the presented target positions and response sizes from the Position-to-Size task, converted from (**a**). (**d**) The relationship between the presented target sizes and response positions from the Size-to-Position task, converted from (**b**). The dashed black lines represent the veridical target sizes and positions. The solid light-colored thin lines are the average responses from each individual participant, where the dots are the means for each simulated target. The darker-colored thick lines show the average slope and intercept from all of the participant responses.

#### Comparing participant responses between tasks

##### Linear model.

The correlation coefficients (*r*) between the stimuli and responses (plotted in Figures 6c and 6d) were between −0.499 and −0.992 (*M* = −0.934, *SD* = 0.106) in the Position-to-Size task and between −0.442 and −0.993 (*M* = −0.922, *SD* = 0.131) in the Size-to-Position task. These values show strong negative linear correlation (|*r*| > 0.6), except for two participants, suggesting a linear relationship. We verified this by fitting a second-order polynomial regression model to the combined response data from all participants. The results from the quadratic component for the Position-to-Size task were not statistically significant, β = 2.59, *SE* = 7.12, *t* = 0.36, *p* = 0.717, but the first-order linear component exhibited a significant negative effect, β = −24.91, *SE* = 5.06, *t* = −4.92, *p* < 0.001. Similarly, for the Size-to-Position task, the quadratic component was not statistically significant, β = 7.52, *SE* = 8.48, *t* = 0.89, *p* = 0.376, but the first-order linear component showed a significant effect, β = −1.82, *SE* = 0.74, *t* = −2.46, *p* < 0.001. When fitting individual participant data, only four participants (out of 40) showed statistical significance (*p* < 0.05) in the quadratic components for both the Position-to-Size and the Size-to-Position tasks, suggesting that the quadratic terms did not significantly improve model fit. Hence, we proceeded to fit the data with a linear model.

A *t*-test comparing average estimated intercepts for each participant between tasks (collapsed across viewing conditions) did not reach significance, *t*(38) = 1.637, *p* = 0.110, Cohen's *d* = 0.262. When compared with the correct intercept value (0.015), using one-sample *t*-tests, the intercepts for both the Size-to-Position task, *M* = −0.003, *SD* = 0.044, *t*(38) = −2.558, *p* = 0.015, Cohen's *d* = −0.410, and the Position-to-Size task, *M* = 0.003, *SD* = 0.033, *t*(38) = −2.340, *p* = 0.025, Cohen's *d* = −0.375, were significantly different. The results from analyzing intercepts are shown in Figure 7a.

**Figure 7.**
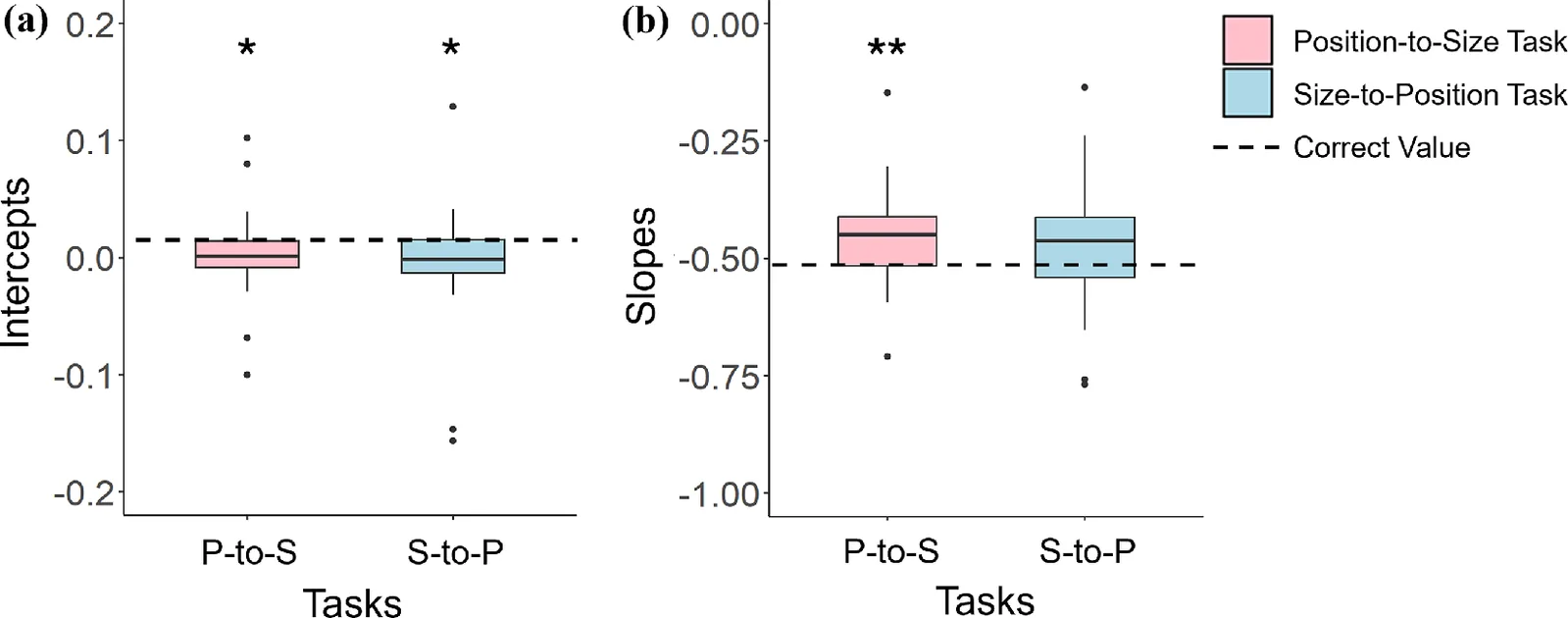
Intercepts and slopes computed from fitting the data with the linear model in Experiment 1. (**a**) The box plots show the mean intercepts from participant responses compared between tasks (Position-to-Size task and Size-to-Position task). These intercepts were also compared with the correct intercept (0.015) from the model. (**b**) The box plots show the mean slopes from participant responses compared between tasks. These slopes were also compared to the correct slope (0.515) from the model. The dashed lines are the correct intercept for (**a**) and the correct slope for (**b**). *p < 0.05, **p < 0.01, ***p < 0.001.

A *t*-test comparing average estimated slopes for each participant between tasks (collapsed across viewing conditions) also did not reach significance, *t*(38) = 1.076, *p* = 0.289, Cohen's *d* = 0.172. Compared to the correct slope (0.515), using one-sample *t*-tests, slopes from the Size-to-Position task (*M* = 0.474, *SD* = 0.129) were not significantly different, *t*(38) = 1.993, *p* = 0.053, Cohen's *d* = 0.319, but the slopes from Position-to-Size task (*M* = 0.461, *SD* = 0.104) were, *t*(38) = 3.271, *p* = 0.002, Cohen's *d* = 0.524. The results from analyzing slopes are shown in Figure 7b.

##### Horizon-ratio model.

Repeated-measures ANOVA revealed no significant main effect of task or distance or interaction between them in the horizon ratios from the participant responses. Because there was no significant effect of any of the variables, we averaged the horizon ratios from each participant across all conditions. One-sample *t*-tests comparing the average horizon ratios (*M* = 0.309, *SD* = 0.091) with the correct ratio in the experiment (0.287) showed no significant difference, *t*(38) = 1.508, *p* = 0.140, Cohen's *d* = 0.242. See Figure 8 and Table C3 for the results.

**Figure 8.**
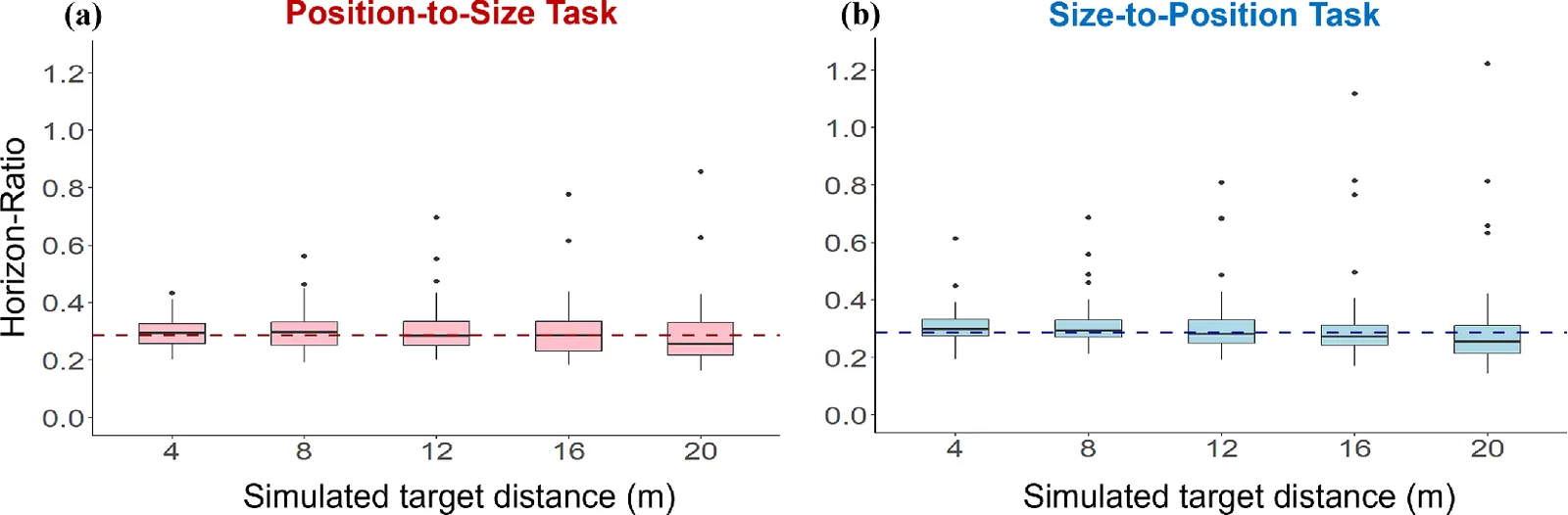
Horizon ratios computed for each experimental condition in Experiment 1. (**a**) The box plots show horizon ratios from the Position-to-Size task. (**b**) The box plots show horizon ratios from the Size-to-Position task. The dashed lines in both (**a**) and (**b**) represent the correct ratio (0.287).

**Table C3.** Repeated-measures ANOVA results for Experiment 2, comparing horizon ratios between relevant conditions.

Variable	*SS*	*df*	*Mean square*	*F*	*p*	$\eta_{p}^{2}$
Task	0.017	1	0.017	0.819	0.371	0.021
Error	0.779	38	0.021	—	—	—
Distance	0.008	1.197	0.006	0.341	0.602	0.009
Error	0.844	45.496	0.019	—	—	—
Task × distance	0.003	1.204	0.002	0.223	0.684	0.006
Error	0.432	45.759	0.009	—	—	—

### Discussion of Experiment 1

Comparing participant responses to the veridical size and position showed that, although the participants accurately positioned targets in the Size-to-Position task, they made the target too large in the Position-to-Size task, as we hypothesized (i.e., larger errors in the Position-to-Size task than in the Size-to-Position task). These results suggest that they could correctly judge target size, leading them to position the target veridically in the scene for the Size-to-Position task. In the Position-to-Size task, the distance cues provided were clearly not as useful for judging target position (or absolute distance) correctly.

The ANOVA results showed that participant responses in both tasks were independent of task order and viewing condition (i.e., whether the scene was viewed monocularly or binocularly). Responses depended strictly on the simulated distance of the target. Therefore, the results provided no evidence to support our hypothesis that people are more accurate in size judgment than distance judgment when viewing monocularly compared to when viewing binocularly, at least when the horizon position is aligned with the viewer's eye level. Interestingly, despite the fact that binocular vision could not help in any way in determining the depicted distance of objects presented in a 2D scene, and even provided conflicting information from the screen itself, participants reported it required less mental load when doing the tasks binocularly compared with doing them monocularly. This result may be due to participants not being used to viewing an object with just one eye.

#### Differences between tasks

We found no statistically significant difference between tasks when we analyzed our data using linear and horizon-ratio models. The average horizon ratios also did not significantly differ from the correct ratio for the reference size and the elevation of the horizon in the scene in this experiment. The horizon-ratio relation has been shown to be effective in determining relative sizes in a picture in the past studies (Bertamini, Yang, & Proffitt, 1998; Rogers, 1996; Sedgwick, 2021). These results may suggest that participants could do the tasks without considering their simulated absolute distances by instead using the horizon ratio. However, based on our analysis, the targets were still made larger than the veridical sizes in the Position-to-Size task. The statistically non-significant differences in the horizon ratios may be due to high between-subject variability or overall noisy responses (see Figure 6c). What possible factors could lead to the targets being made too large?

##### Misperceived target position.

Participants focusing on different parts of the target may have affected their responses in the two tasks. When adjusting the target size, the participants had to attend to the top (as the bottom of the target was fixed) but when they were adjusting its position they likely attended to the bottom of the target (where it touched the ground). Participants focusing on the top of the target may have led to errors when judging its position on the ground (the bottom of the target), overestimating its angular declination and resulting in them making the target too large. However, whether focusing on the top of the object during the task or focusing on different parts of the object (i.e., the top or the bottom) affects people's perception of the position of an object has yet to be verified.

##### Misperceived size.

Either underestimating the visual size of the target or overestimating the haptic size of the felt reference box could lead to needing to make the target larger to match it to the reference box in the Position-to-Size task. However, the participants correctly placed targets in the scene based on the target sizes in the Size-to-Position task. They should not have been able to do this if either target or reference size was misperceived. The correctly gauged horizon ratios from participant responses also suggest it is unlikely that the targets were made too big due to misperceiving their size. Could such specific errors arise from misperceiving the horizon or their eye height?

##### Misperceived horizon or eye height.

The intercepts and slopes estimated from the linear model fit represent the elevation of the horizon in a 2D scene and the observer's eye height, respectively (refer to Appendix A for details). In our analyses, we assumed the perceived horizon and eye height from the computed intercepts and slopes (see Figure 7). The significantly smaller slopes in the Position-to-Size task may suggest that the participants underestimated their eye height. Such a misperception could arise either from them thinking that their eye level was lower or the ground was higher than it really was. However, the scene presented in this experiment was identical in both tasks, and it is unlikely that the perceived eye height depended on task. Also, the slopes did not significantly differ between tasks.

The intercept results suggested that the perceived horizon may have been below the actual horizon position in the scene in general. An artificially lowered horizon has been shown to influence people's perceived distance in virtual reality (Messing & Durgin, 2005). The misperceived horizon in our scene (e.g., leading to an incorrectly computed horizon ratio) may have led to the errors in our participants’ perceived absolute distance. However, the perceived horizon and eye heights in Experiment 1 were estimated indirectly using linear model fitting; hence, they may not be an accurate estimation from which to make solid conclusions. People's perceptions of the horizon position in a 2D scene and their eye height should be measured directly using a more comprehensive method.

#### Difference in display size

Considering the results from the past study (Kim & Harris, 2022), where people could not accurately derive absolute distance from the size or position of an object in a 2D scene, the visual stimuli and setup used in Experiment 1 seem to have improved participant performance. (The differences in the results between the experiments, as well as the possible contributing factors that led to the improvement shown, are highlighted in Appendix D.) The biggest change made with Experiment 1 was the display size, where the scene was presented on a large projection instead of on a small monitor screen. Therefore, in Experiment 2 we used a separate monitor where the scene was the same as on the projection. Such a design allowed us to evaluate whether the difference in participant responses was due to them misperceiving the elevation of the horizon in the scene or misjudging their eye level as a result of the different display sizes.

## Experiment 2

In Experiment 2, participants aligned a horizontal bar on the screen with either their eye level (eye level task) or the horizon position in the scene (horizon task). The experiment was a 2 × 3 design with *displays* and *scenes* as the within-subject factors. The displays were projection and monitor. The presented scene was the same in both displays with identical visual angles of the key features (e.g., top and bottom of the scene and the side walls). The scenes were low-horizon (the horizon in the scene was lower than the participant's eye level), middle-horizon (the horizon and the eye level were aligned), and high-horizon (the horizon was higher than the eye level) scene conditions. The participants’ eye level was kept constant in all conditions. We hypothesized that (a) people would place the horizontal bar in the eye-level task lower when viewing the scene on a monitor than on a projection, and (b) people would place the horizontal bar in the horizon task closer to their eye level when the horizon and the eye level were misaligned (i.e., lower in the high-horizon scene condition and higher in the low-horizon scene condition).

### Methods for Experiment 2

#### Participants

Fourteen undergraduate students (nine females and five males; average age, 21.8 ± 5.29 years) participated in the experiment in the period between Jun 4, 2025, and August 7, 2025. After our data cleaning process, no participant was removed from the analysis. All participants were students recruited via the URPP from York University, Toronto, ON, Canada. They had normal or corrected-to-normal vision and signed an informed consent form before participating. Students were given course credit for their participation. The experiments were approved by York University's Ethics Review Board and conducted in accordance with the relevant guidelines and regulations.

#### Experimental setup and stimuli

The visual stimuli were modified versions of the hallway scene from Experiment 1 and were either projected onto a wall (Figure 2a) or on a computer monitor (Dell Ultra HD 4K P2715QT 27-inch LED monitor with in-plane switching [IPS], turned vertically) (Figure 9a). Real carpet was not used in Experiment 2 to match the visual setup of the projection to the setup of the monitor.

**Figure 9.**
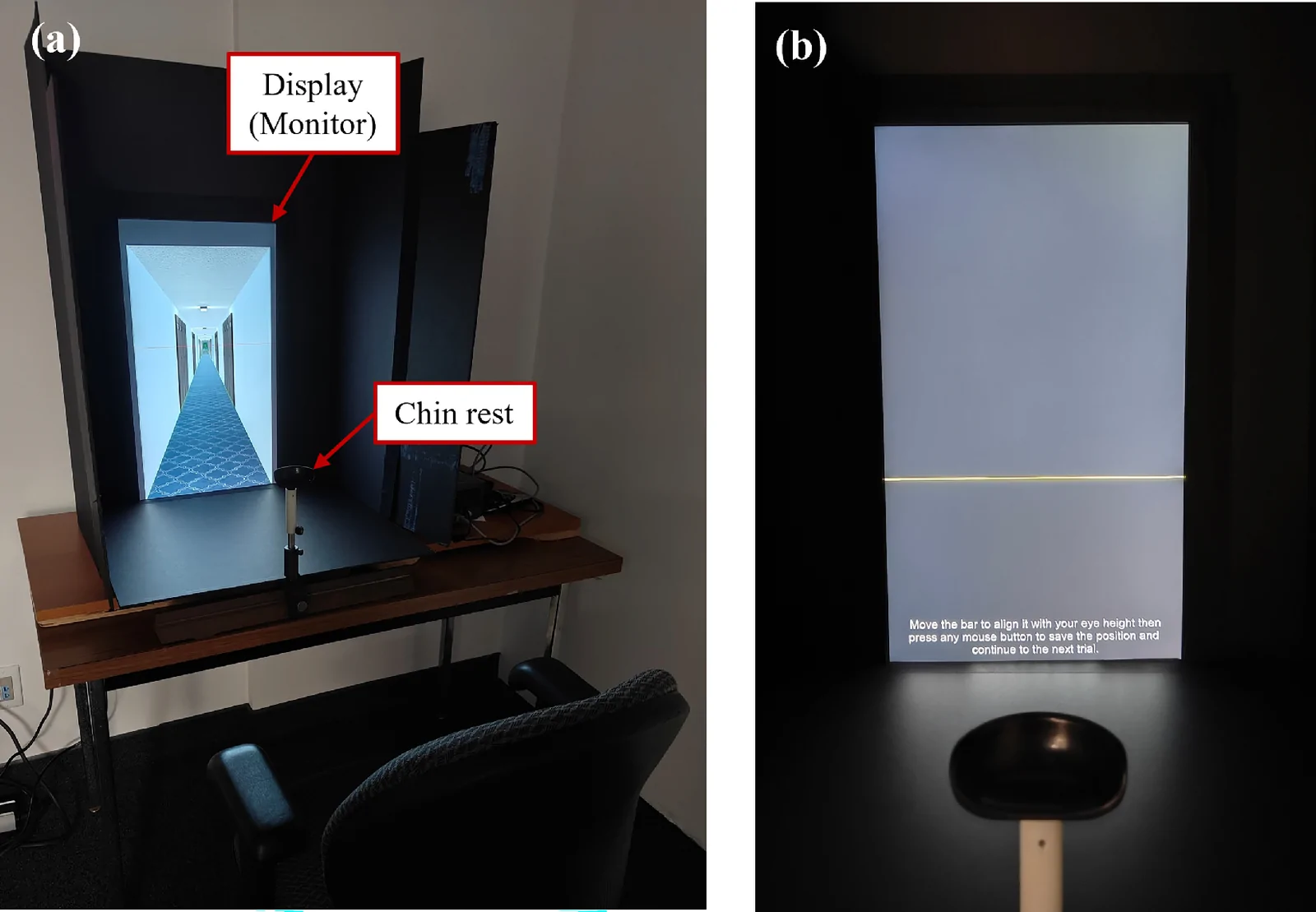
Apparatus and monitor setup in Experiment 2. (**a**) The experimental setup for the monitor conditions. The distance between the eyes to the display was 0.5 meter (50 cm) when the participant's chin was on the chin rest. (**b**) An example of response phase. Participants moved the horizontal bar, using the hand-held mouse, to align it with either the horizon or their eye level.

The visual angles of the features presented on both displays were aligned (see Figure 10a). There were three scenes with different horizon heights. In each scene, the horizon of the scene (i.e., the vanishing point) was positioned 5° below the eye level (low) (Figure 10b), aligned with the eye level (middle) (Figure 10c), or 5° above the eye level (high) (Figure 10d). The eye level of the participant was consistent between conditions.

**Figure 10.**
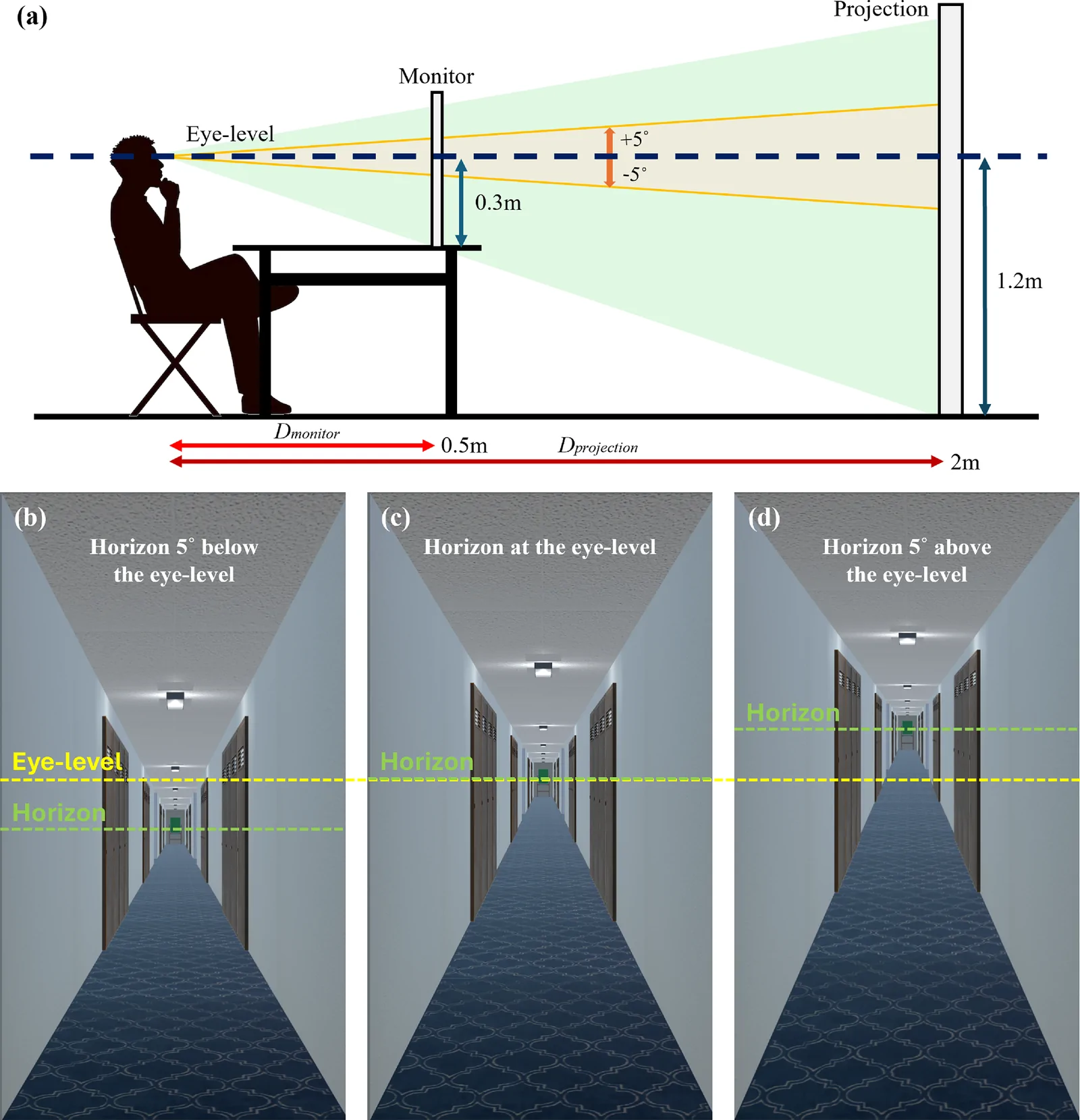
The visual stimuli presented on the two displays. (**a**) The visual angles of the scene: the bottom and the top of the scene and the horizons (−5°, 0°, and +5° from eye level) aligned between the projection (2 meters away) and the monitor (0.5 meter away). (**b**–**d**) Three scenes were used with varying horizon height: low (**b**), middle (**c**), and high (**d**).

#### Procedure

Before the experiment, it was explained to the participants what the horizon is and they were shown some example images of indoor and outdoor scenes with the horizon visible or hidden. Then, they were given verbal instructions for the experimental tasks (see below). After the instruction, they sat on a chair in the test room with their chin on the chin rest. To verify our setup assuming the distance between the chin to pupils as 0.12 m, we measured this distance for each participant. The mean distance was 0.117 ± 0.007 meter.

All participants did the experiment on both the projection and the monitor displays, one following the other. The order of the displays in the experiment was counterbalanced between participants. At the beginning of the experiment on each display, before viewing any of the scenes on it, each participant aligned a horizontal bar (shown in Figure 9b) with their visually perceived eye level (VPEL) five times using a hand-held mouse (Figure 2b). This measured the baselines of their VPEL for each display.

Each trial started with a display of static visual white noise for 1 second to indicate the start of a trial, followed by the hallway scene for 1 second. After the scene disappeared, participants saw a horizontal bar and did one of two experimental tasks: eye-level task (align the yellow horizontal bar with their VPEL) or horizon task (align the green horizontal bar with the horizon in the previously presented scene). When the bar was aligned with either the eye level or the horizon to their satisfaction, the participant pressed a button on the hand-held mouse to continue to the next trial. The bar positions were recorded as between −0.5 (bottom of the screen) and +0.5 (the top of the screen) where the true eye level was 0.

The experiment was comprised of 60 trials (5 trials × 2 tasks × 6 blocks) on each display (two displays) totaling 120 trials (not counting the initial eye level measurements for each display). Each block consisted of the eye-level task (five trials) followed by the horizon task (five trials), with one following the other (i.e., all five trials for each task were lumped together), or vice versa. The order of the tasks was counterbalanced between blocks. The experiment took approximately 45 minutes to complete.

#### Data analysis

Two separate repeated-measures ANOVAs were conducted. Each ANOVA compared the positions of the bar from the eye-level task (*P_eye_*) or the horizon task (*P_horizon_*) between the displays (projection and monitor) and the scenes (low, middle, and high). The averages were also compared with their correct position on the screen using *t*-tests to evaluate accuracy. A family-wise error rate of 0.05 was used for all analyses controlled with the Holm–Bonferroni method for adjusting *p* values in multiple comparisons. For any ANOVA violating the assumption of sphericity, the degrees of freedom were adjusted using the Huynh–Feldt correction, as the estimated epsilon (ε) was generally less than 0.75.

##### Data cleaning process

We considered any values outside the range of the mean (±2.5 × *SD*) in each condition as outlying data points and removed them from the dataset prior to the analysis. This data cleaning process removed no data points from the total dataset, and no participant had to be removed from the analysis.

### Results of Experiment 2

#### Perceived horizon

A repeated-measures ANOVA, conducted to compare *P_horizon_* between displays and scenes showed a significant main effect of scene, *F*(1*.*478, 19*.*210) = 897.379, *p* < 0.001, $\eta_{p}^{2}$ = 0.986. There was no effect of display, and no interactions were found. Further evaluation revealed that *P_horizon_* values were lowest with the low-horizon scene and highest with the high-horizon scene: low (*M* = −0.073), middle (*M* = −0.012), and high (*M* = 0.058).

We compared *P_horizon_* with the correct horizon position (i.e., −0.073 for low, 0 for middle, 0.073 for high) for each scene using one-sample *t*-tests (one-tailed). The analysis, which corrected the *p* values using the Holm–Bonferroni method, revealed that the horizon was judged significantly lower than the correct position in the high-horizon scenes (*p* = 0.006, Cohen's *d* = 0.880) when viewed on a monitor (see Figure 11a).

**Figure 11.**
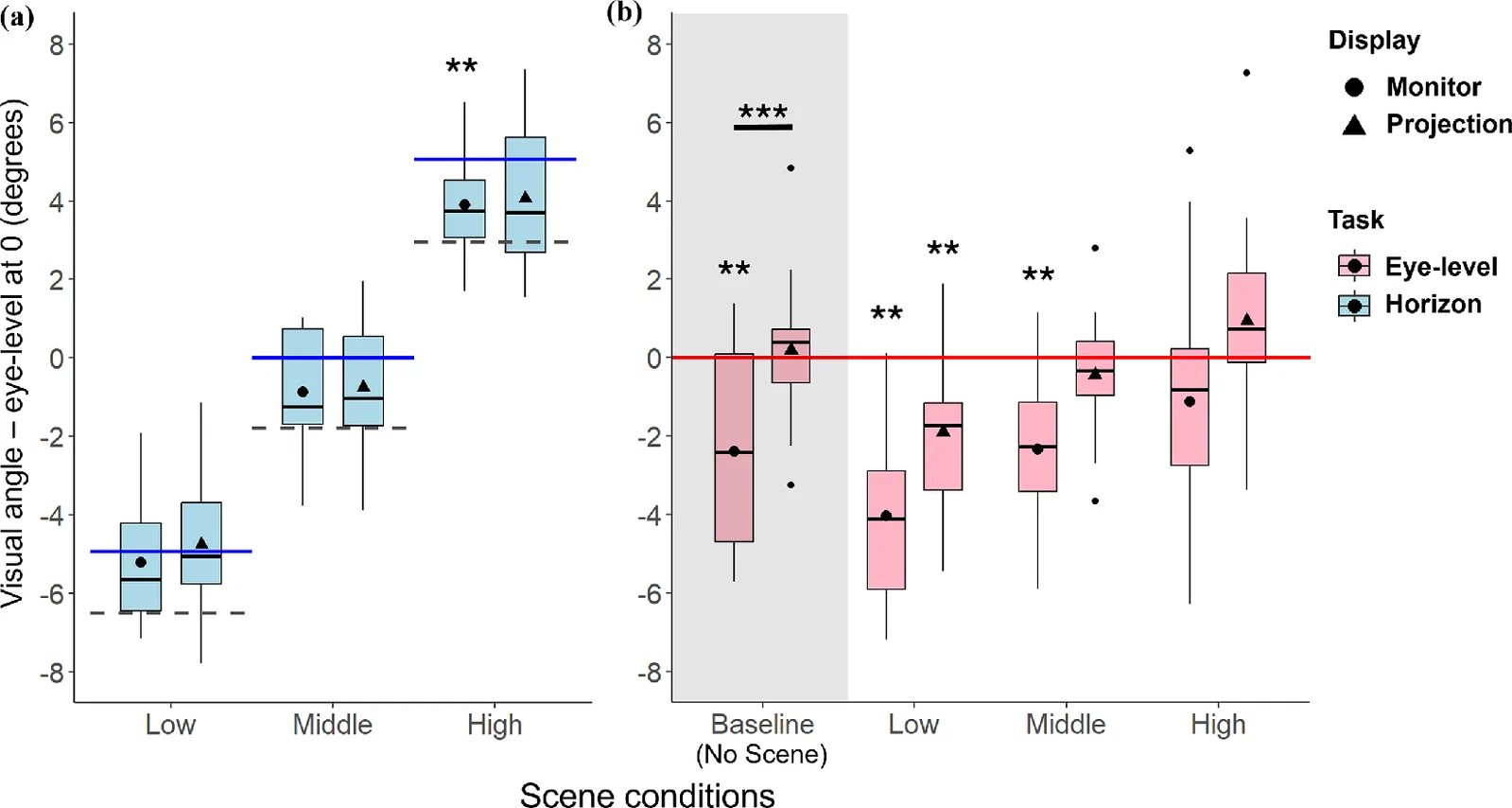
Horizon (*P_horizon_*) and eye-level (*P_eye_*) results expressed as visual angles for Experiment 2. Participant responses from each experimental condition are plotted as a boxplot with each point (circle, monitor; triangle, projection) representing the average. (**a**) The boxplots show the horizon results (in light blue) with the solid lines (in blue) representing the correct horizon for each scene condition. The dashed lines (in black) represent where the end of floor was in each scene. (**b**) The box plots show the eye height results (in pink) with the solid line (in red) representing the correct eye height. ∼*p* < 0.10, *p < 0.05, **p < 0.01, ***p < 0.001.

#### Visually perceived eye level

A repeated-measures ANOVA comparing *P_eye_* between the experimental conditions showed significant main effects of display, *F*(1, 13) = 23.304, *p* < 0.001, $\eta_{p}^{2}$ = 0.642, and scene, *F*(1*.*080, 14*.*045) = 10.784, *p* = 0.005, $\eta_{p}^{2}$ = 0.453. No significant interaction was found. Further evaluations revealed that *P_eye_* values were lower (closer to the ground) when experiments were performed on the monitor than they were for the wall projection. There were also significant differences between low and middle scenes (*p* = 0.008) and middle and high scenes (*p* = 0.017).

We compared *P_eye_* to the correct eye level (i.e., 0) using one-sample *t*-tests (one-tailed). The analysis, which corrected the *p* values using the Holm–Bonferroni method, revealed that *P_eye_* was significantly lower than the correct height when the horizon in the scene was in the middle (*p* = 0.002, Cohen's *d* = 1.016) or low (*p* < 0.001, Cohen's *d* = 1.824) when viewed on the monitor and when the horizon was low (*p* = 0.005, Cohen's *d* = 0.903) when viewed on the projection. The baseline *P_eye_* values (i.e., without viewing any scene) were also too low when participants did the task on the monitor (*p* = 0.003, Cohen's *d* = 0.967) but not when they did it on the projection (*p* = 0.721, Cohen's *d* = 0.098). When comparing the results between the displays, the baseline *P_eye_* was significantly lower on the monitor compared with when it was measured using the projection, *t*(13) = 5.581, *p* < 0.001, Cohen's *d* = 1.491 (see Figure 11b).

### Discussion of Experiment 2

The significant effect of display on VPEL in the baseline condition of the eye-level task, where no scene was displayed, showed that the participants thought their eye level was lower when they were looking at the small monitor screen than the full-size projection, but this display-dependent difference did not reach significance when the hallway scene was displayed on the screen. Therefore, our hypothesis that the eye level would be judged as lower on a monitor than on a projection was only partially supported.

Participants accurately aligned the horizontal bar with their eye level when they viewed the scenes on the projection (see Figure 11). The errors in their perceived horizon position only reached statistical significance for the middle-horizon scene conditions on the monitor, where participants placed the bar lower than the horizon in the scene (i.e., closer to their eye level). However, other than this one condition, the errors did not significantly differ from the correct horizon position; therefore, our hypothesis that people would assume the horizon was aligned with their eye level was not supported.

#### Perceiving the horizon in a 2D scene

No significant differences between displays were found in any scene conditions, which indicates that the display size did not impact participants’ perception of the horizon position in the scene. Participants, however, underestimated the elevation of the horizon in the 2D scenes in general. Such results may be due to the real horizon not being explicitly visible. People's visually perceived distance (Lappin, Shelton, & Rieser, 2006; Teghtsoonian & Teghtsoonian, 1969; Witt, Stefanucci, Riener, & Proffitt, 2007) and horizon position (Ozkan & Braunstein, 2010) are influenced by the environmental context such as its surface structure. Our participants may have been influenced by such a visual cue, such as the edge between the wall and the floor at the end of the hallway (represented as black dotted lines in Figure 11a). In some cases, the bars were placed even lower than the artificial horizon formed by the visible end of the corridor. Such responses may indicate that participants thought that the hallway was slanted upward. It could also be influenced by the scene being only visible for a short period of time. One second may not be enough time for a participant to fully gauge the horizon in a complex scene where the horizon is not actually clearly visible.

The horizon in a scene, whether real or an image, can be determined from the vanishing point, which can be gauged using various visual cues (Bellutta, Collini, Verri, & Torre, 1989). Such cues are fairly obvious when looking down a corridor with parallel walls. Our results demonstrated, contrary to Rogers (1996), that people do not always assume that the horizon in a 2D scene is aligned with their eye level. Rather, people may be quite accurate at judging the elevation of the horizon in a picture regardless of their eye level.

### Perceiving eye level while looking at a screen

The horizon, or the vanishing point, in a scene appears to be an important cue for people when judging their eye level. After seeing the low-horizon scene, participants’ VPELs were lower than their real eye level. The VPELs were slightly higher than their real eye level after seeing the high-horizon scene, although the difference did not reach statistical significance. Participants also showed no display-dependent differences in their VPELs when the hallway scene was presented on the screen. These results suggest that the horizon location in the scenes influenced people's perceptions of their eye level in a 2D scene, as Dixon et al. (2000) suggested.

The horizon in the scene was not the only factor that influenced the participants’ perceptions of eye level. Participants also judged their eye level as lower when looking at a small monitor screen. A possible reason for such an error may be the availability of the ground information. People are quite accurate at judging their eye height when standing in a real corridor (Mark, 1987). Although our participants were seated during both parts of this experiment, they judged their eye level accurately when looking at a projection. In the projection condition, participants could see the ground between their feet and the wall (see Figure 3a, without the real carpet). However, when looking at the monitor they could only see the top of the desk (see Figure 9). A restricted view of the ground has been shown to make the ground surface perceived as slanted upward, leading to distance underestimation (Wu, Ooi, & He, 2004). The lack of ground information may have played a role in the erroneous gauging of eye height, leading to errors in eye-level judgment.

## General discussion

In Experiment 1, we showed that people likely judge relative sizes and distance of objects in a 2D scene by gauging the size and position relationship of the object, such as the horizon ratio, which requires accurately perceiving the elevation of the horizon in the scene (Sedgwick, 2021). Although the linear model fit results from Experiment 1 suggested that people may underestimate the elevation of the horizon in a scene, the errors participants made in Experiment 2 when directly aligning a horizontal line to the horizon in the identical scene presented on projection did not significantly deviate from the true horizon. Such results suggest that people may be accurate at judging horizon position in a 2D scene and therefore effectively use the horizon ratio to judge object sizes.

In Experiment 2, we also demonstrated that the horizon in pictures alters people's VPEL. Therefore, people are likely judging their eye level based on their perception of the horizon position in the scene (per Dixon et al., 2000) rather than judging the horizon position based on their eye level. People judge their eye level as lower when they look at a small screen (e.g., a monitor), even when the horizon and the eye level are properly aligned. This error may be due to the lack of ground information leading to underestimation of their eye height. Misjudged eye height can lead to an object being judged as larger in a virtual environment (Ghasemi, Harris, & Jörges, 2023). Such misperception may, at least partially, be the cause in the suppression of perceived space when viewed on small television (Roscoe, 1984) and the poor performance in our previous study using small monitors (Kim & Harris, 2022). However, relying on eye level is not a reliable method to judge distance in an image where the scene does not always align with the viewer's eyes (as demonstrated in Figure 1). Hence, misperceived eye level cannot fully explain the errors people make when judging size and distance in a 2D scene, such as the targets being made too large in the Position-to-Size task in Experiment 1.

Responses from some participants for the projected scene in Experiment 2, judging the horizon below the ground surface, suggest that they may have perceived the floor as slanted upward and the true horizon was hidden behind it (such as a hill). In such a case, the visual size of an object would have to be larger compared with the object on a level floor, if the angular declinations of the base of the object from the eye level were the same (see Figure 12 for an illustration).

**Figure 12.**
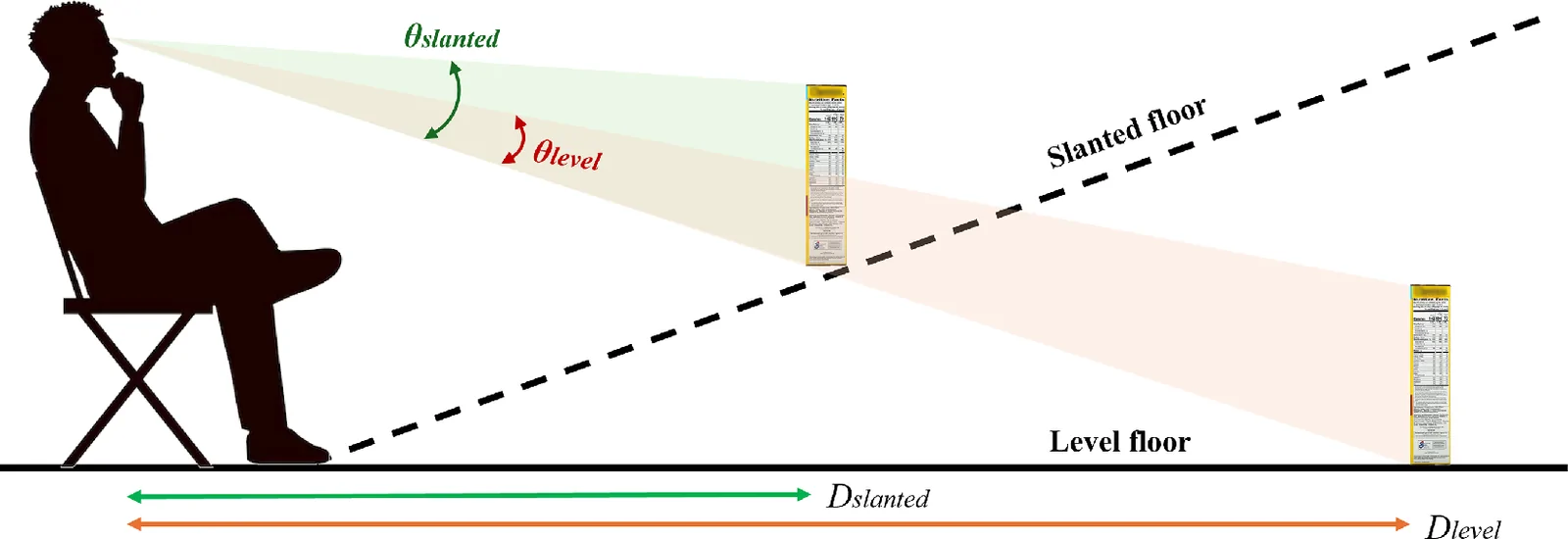
Example of objects on a level or a slanted floor. Objects on a slanted floor viewed by an observer subtend a larger visual angle (θ*_slanted_*) and closer in absolute distance (*D_slanted_*) compared with the object positioned at the identical angular declination on a level floor (with visual angle of θ*_level_* and absolute distance of *D_level_*).

A level, or flat, ground surface can be perceived as slanted when a person's field of view is limited (Wu et al., 2004). To avoid this slant error in Experiment 1, we aligned a real carpet with the virtual carpet in the projected scene. The targets being made too large in the Position-to-Size task may indicate that (a) the real carpet did not provide sufficient ground information, or (b) people focused on what was shown on the projected scene, paying less attention to the real carpet. Further research is necessary to determine which of these possible factors was the most influential, or if perhaps something entirely different contributed to the size errors we found.

Regardless, the misperceived slant on the floor would also impact the Size-to-Position task, and the results from Experiment 1 show clear differences between the two tasks (Size-to-Position and Position-to-Size). Although people were much more accurate at judging the size and position of the objects for both tasks in the present study compared with a past study conducted on computer screens (Kim & Harris, 2022), the change in the experimental setup (i.e., presenting the 2D scene as a large projection on a wall with near-ground information) seems to have impacted the two tasks differently. Such findings further support the idea that size and distance perception may use separate brain mechanisms (Haber & Levin, 2001; Kim, 2018; Kim & Harris, 2022; Mon-Williams & Tresilian, 1999; Ozkan & Braunstein, 2010).

## Conclusions

Although people can easily recognize and perceive objects in 2D images or pictures of a scene, they may not correctly perceive the 3D space represented by the scene they are looking at. Overall, our results revealed that caution is needed when picking a background scene when testing people's size or distance perception ability. People generally judge their eye level as lower when looking at a small screen such as a monitor, possibly due to insufficient ground information. Their perceived eye levels are also influenced by the perceived horizon positions, which may be influenced by the clearly visible horizontal lines (in the case of the present study, the edge between the wall and the floor). Therefore, for people to correctly judge spatial properties of 2D scene and the objects in the scene (e.g., size and distance), (a) the horizon in the scene and the viewer's eye level must be aligned, and (b) the scene must be presented on a large immersive display. The correct judgment is likely from the convincing nature of the scene displayed on a larger screen, allowing the person to correctly judge their eye height from correctly perceiving their eye level.

### Transparency and openness

We report all data exclusions, all manipulations, and all measures in the study, and we followed APA Style Journal Article Reporting Standards (Appelbaum, Cooper, Kline, Mayo-Wilson, Nezu, & Rao, 2018). All data, analysis code, and research materials are available at https://osf.io/9xt7f/. Data were analyzed using JASP 0.19.3.0, and the plots were made using R 4.2.2 (R Core Team, 2022) and the ggplot 2 3.5.2 package (Wickham, 2016). The design of this study and its analysis were not pre-registered.

## Figures and Tables

**Figure A1.**
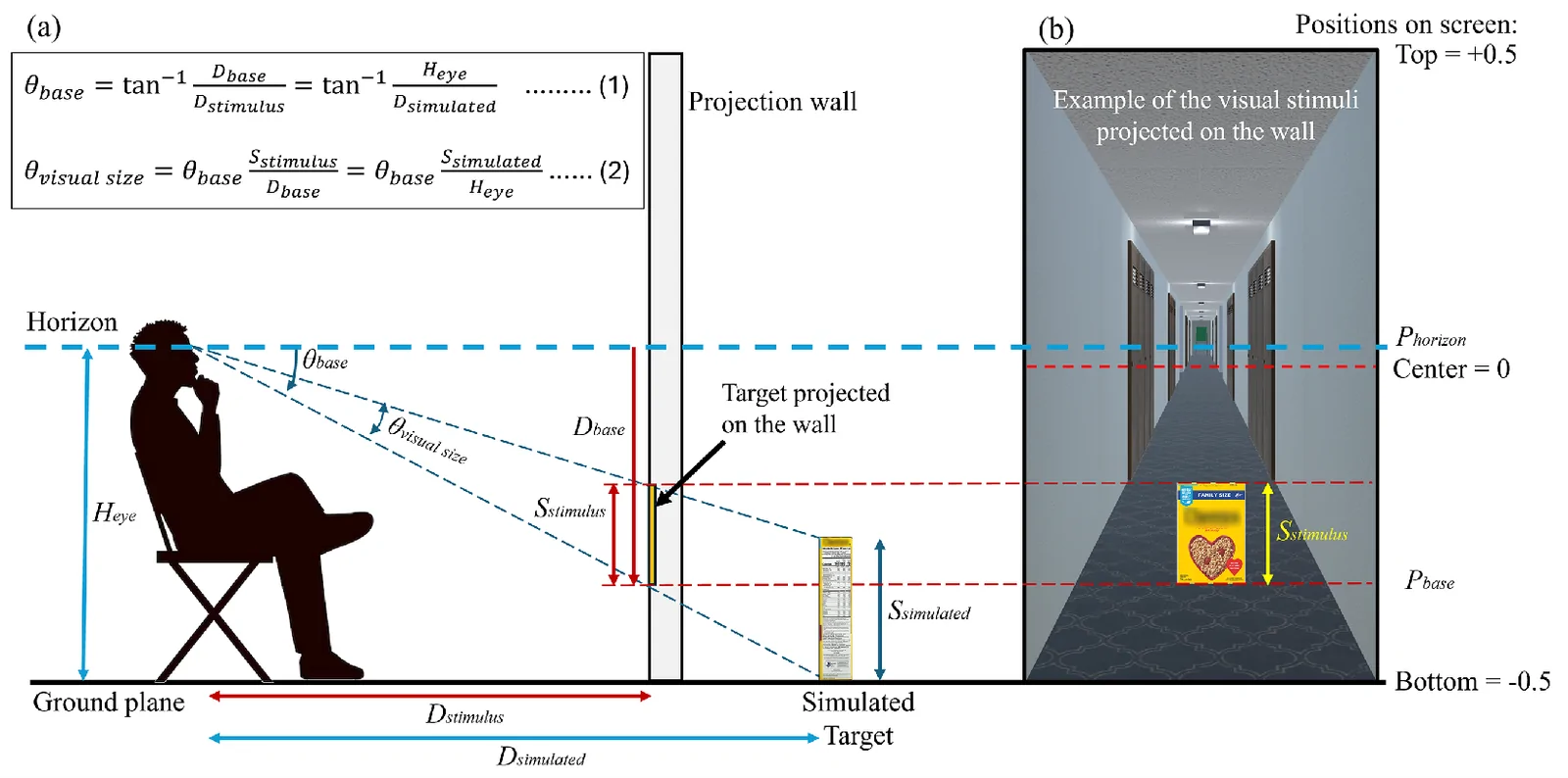
Example of a target distance simulated in a projection. (**a**) Diagram shows the geometry and formulas of a target, simulated to be at a set distance away from the observer presented in the projected scene. (**b**) The visual stimuli projected on the wall simulating the target from panel (**a**).

**Figure A2.**
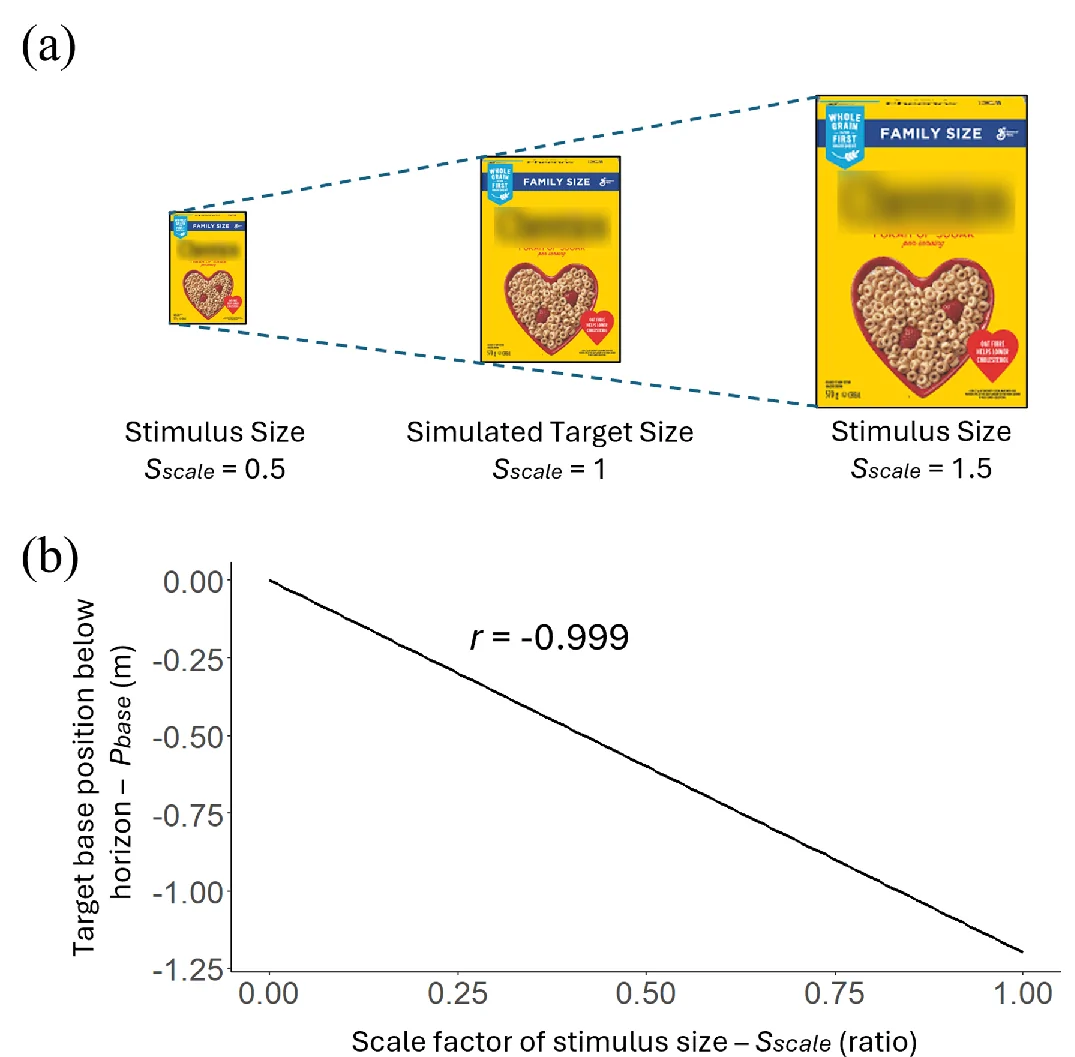
(**a**) Some examples of stimulus size (*S_stimulus_*) in relation to the simulated size (*S_simulated_*) using the scale factor (*S_scale_*). (**b**) The veridical relationship between stimulus size (*S_scale_*) and target base position (*P_base_*). The correlation coefficient of *r* = −0.999 represents very strong linear relationship between them.

**Figure D1.**
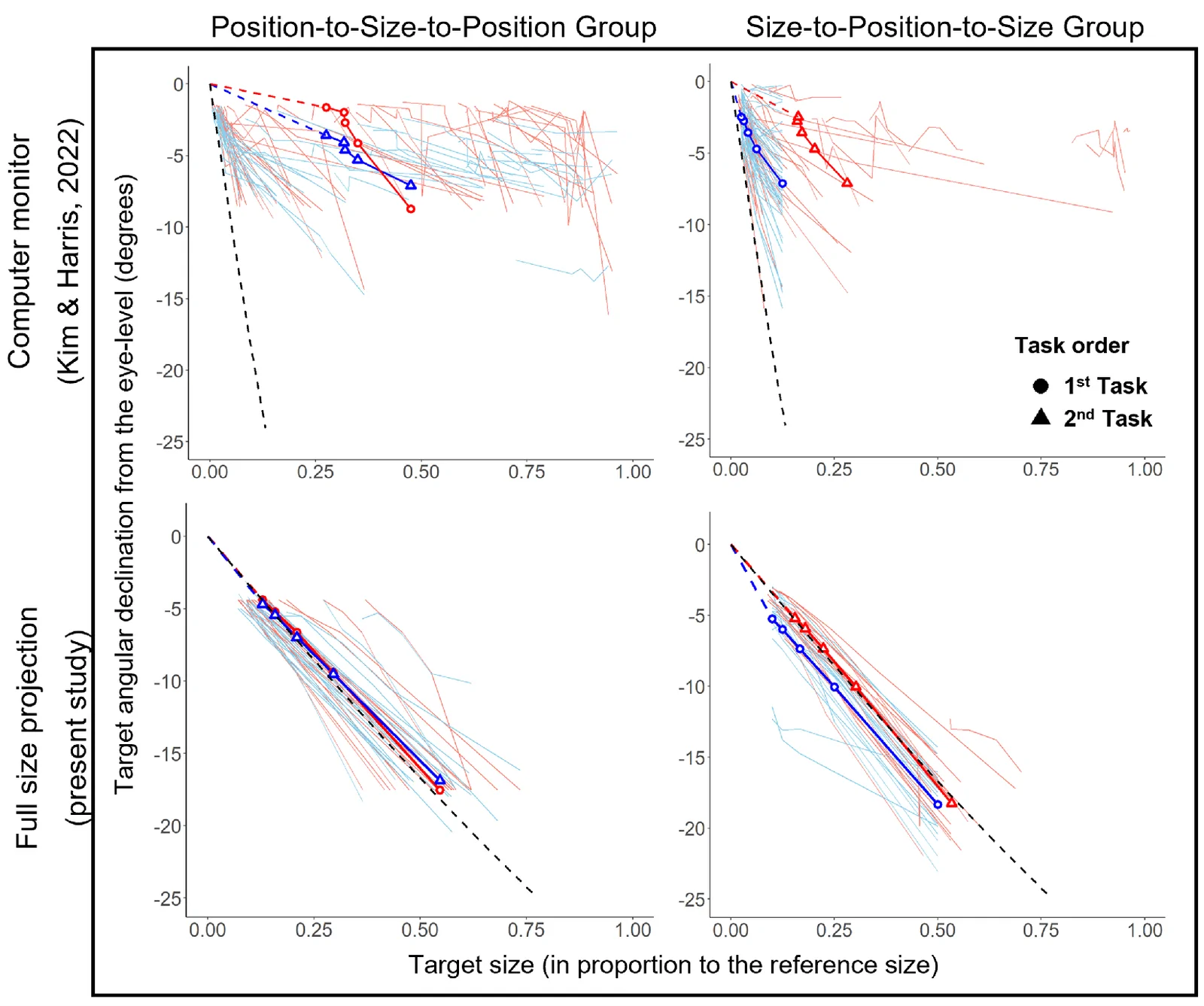
Participants’ responses in the past and the present experiments. Target positions are plotted as a function of target size. The top row is from the past experiment where the experiment was conducted online on computer screens (Kim & Harris, 2022). The bottom row is from Experiment 1, where the experiment was conducted in lab on a full-scale projection separated by the task order. (Left column) The Position-to-Size task first followed by the Size-to-Position. (Right column) The Size-to-Position task first followed by the Position-to-Size task. The dashed black lines represent target sizes and positions with the correct geometry. The solid light-colored lines are the average responses from each individual participant, and the darker colored lines with white dots represent the mean of responses from all participants for each task, where the dots are the means for each simulated target: Size-to-Position task (blue) and Position-to-Size task (*red*).

## Appendix A: Appendix A: Linear relationship between object size and position in a picture

The distance of an object seen by an observer can be determined from a combination of its position on the screen (the visual declination of its base, θ*_base_*) and its size (angular size of the object, θ*_visual size_*), computed by Equations A1 and A2 in Figure A1. This object of fixed size (*S_simulated_*) on the ground at a fixed distance (*D_simulated_*) can be simulated on a 2D screen (as shown in Figure A1b) following the geometry in Figure A1a. In this simulated scene, the position of the object (distance below the horizon, *D_base_*) and its size (*S_stimulus_*) on the screen depend on the distance of the screen from the observer (*D_stimulus_*). The position and the size of the object vary in accordance with its simulated distance (*D_simulated_*). Specifically, *S_stimulus_* becomes larger with closer *D_simulated_* and smaller with farther *D_simulated_*. *D_base_* becomes longer with closer *D_simulated_* and shorter with farther *D_simulated_*.

In our experimental setup, *D_stimulus_* was 2 meters. The horizon was at the observer's eye height (*H_eye_*), or eye level. When presenting a target, its position was determined by its base position on screen (*P_base_*), which was based on the horizontal length of the screen with its full length being 1. The center position was set to 0, the top of the screen was +0.5, and the bottom of the screen was −0.5 (refer to Figure A1b). In the scene, the position of horizon (i.e., the eye level) was at 0.015; therefore, the eye height was 0.515 (the distance between the horizon and the bottom of the screen).

The stimulus size (*S_simulated_*) of the object on screen can be represented as a scale factor (*S_scale_*) which can be between 0 (target not visible) and 1 (full size of the simulated box, or the reference box). Figure A2a illustrates this following Equation A1.

The object position (i.e., its base of the object on screen below the horizon, or eye level), can be computed by

(A3) $$\begin{array}{c} D_{base}=H_{eye}\times \frac{D_{stimulus}}{D_{simulated}}=H_{eye}\times S_{scale}\; \end{array}$$

Therefore, its position (*P_base_*) is

(A4) $$\begin{array}{ccc} & & \;P_{base}=P_{horizon}-D_{base}=P_{horizon}-\left(H_{eye}\times S_{scale}\right)\;\;\; \end{array}$$

With the horizon at 0.015 and eye height of 0.515 in our experimental setup, the relationship between *S_scale_* and *P_base_* can be represented as

(A5) $$\begin{array}{c} P_{base}=0.015-\left(0.515\times S_{scale}\right)\; \end{array}$$

Equation A5 is verified by the strong linear relationship (Pearson's *r* = −0.999) between the veridical object position and size on a screen, plotted in Figure A2b.

## Appendix B: Appendix B: NASA TLX

On each scale, write the number that best reflects your experience with the task.

### Question 1

How much mental load was required for understanding and processing information (e.g., thinking, deciding, calculating, remembering, looking, searching, ...)? Was the task easy or demanding, simple or complex, does it require high accuracy or is it fault-tolerant?

*Mental requirements*: 1 (low) to 7 (high)

### Question 2

How much physical activity was required (e.g., pulling, pressing, turning, controlling, activating, …)? Was the task easy or difficult, easy or exhausting, restful or tedious?

*Physical demands*: 1 (low) to 7 (high)

### Question 3

How much time pressure did you feel in terms of the frequency or rhythm with which the tasks or task elements occurred? Was the task slow and leisurely or fast and hectic?

*Time requirement*: 1 (low) to 7 (high)

### Question 4

In your opinion, how successfully have you achieved the goals set by the experimenter (or yourself)? How satisfied were you with your performance in pursuing these goals?

*Achievement*: 1 (good) to 7 (bad)

### Question 5

How hard did you have to work to achieve your level of task accomplishment?

*Effort*: 1 (low) to 7 (high)

### Question 6

How insecure, discouraged, irritated, stressed, or upset (vs. safe, confirmed, satisfied, relaxed, and satisfied with yourself) did you feel during the task?

*Frustration*: 1 (low) to 7 (high)

## Appendix D: Appendix D: Comparisons between past and present studies

As shown in Figure D1, in a past study (Kim & Harris, 2022) the target sizes were made bigger than the veridical values on average in the Position-to-Size task. The target positions were also made higher on average (closer to the horizon) in the Size-to-Position task, but those errors were much smaller in Experiment 1, showing that they were much more accurate at the tasks in general. The participant responses in the past study were also much noisier and deviated further from the correct answers than Experiment 1. Various factors may have contributed to this improvement.

1. Although the participant pool was identical (undergraduate students from York University), the past study was conducted online. The noisy responses in the past study may be due to the reduced control over the experimental settings (e.g., monitor sizes) and supervision in an online study. Experiment 1 was conducted in-lab where the experiment was more carefully controlled.
2. Human perception can be influenced by a person's cognitive state of thinking whether something is real or not (D'Amour, Harris, Berti, & Keshavarz, 2021; Interrante, Ries, & Anderson, 2006; Kim & Harris, 2024). A more realistic visual scene in Experiment 1 compared to the past study (e.g., an indoor rather than an outdoor scene and a life-sized scene projection rather than a screen-sized scene on a monitor) may have affected people's interpretations of visual stimuli at a higher cognitive level (e.g., from their previous experience or memory), resulting in better spatial perception.
3. Finally, the display size differed where the past study was completed on a small computer monitor, although it could not be controlled due to the study being conducted online, but Experiment 1 used a large projection on a wall.

## References

[bib1] Appelbaum, M., Cooper, H., Kline, R. B., Mayo-Wilson, E., Nezu, A. M., & Rao, S. M. (2018). Journal article reporting standards for quantitative research in psychology: The APA Publications and Communications Board task force report. *American Psychologist,* 73(1), 3. 10.1037/amp0000191 29345484

[bib2] Bellutta, P., Collini, G., Verri, A., & Torre, V. (1989). 3D visual information from vanishing points. In *Proceedings: Workshop on Interpretation of 3D Scenes* (pp. 41–49). Piscataway, NJ: Institute of Electrical and Electronics Engineers.

[bib3] Bertamini, M., Yang, T. L., & Proffitt, D. R. (1998). Relative size perception at a distance is best at eye level. *Perception & Psychophysics,* 60(4), 673–682. 10.3758/bf03206054 9628998

[bib4] Brenner, E., & Smeets, J. B. (2018). Depth perception. In J. T. Wixted (Ed.), *Stevens’ handbook of experimental psychology and cognitive neuroscience: Sensation*, *perception*, *and attention* (pp. 385–414). New York: Wiley.

[bib5] Brenner, E., & van Damme, W. J. (1999). Perceived distance, shape and size. *Vision Research,* 39(5), 975–986. 10.1016/s0042-6989(98)00162-x 10341949

[bib6] D'Amour, S., Harris, L. R., Berti, S., & Keshavarz, B. (2021). The role of cognitive factors and personality traits in the perception of illusory self-motion (vection). *Attention*, *Perception*, *& Psychophysics,* 83(4), 1804–1817. 10.3758/s13414-020-02228-3 PMC8084801 33409903

[bib7] Dixon, M. W., Wraga, M., Proffitt, D. R., & Williams, G. C. (2000). Eye height scaling of absolute size in immersive and nonimmersive displays. *Journal of Experimental Psychology: Human Perception and Performance,* 26(2), 582–593. 10.1037//0096-1523.26.2.582 10811164

[bib8] Durgin, F. H. (2014). Angular scale expansion theory and the misperception of egocentric distance in locomotor space. *Psychology & Neuroscience,* 7, 253–260. 10.3922/j.psns.2014.032 PMC4297657 25610539

[bib9] Ghasemi, F., Harris, L. R., & Jörges, B. (2023). Simulated eye height impacts size perception differently depending on real-world posture. *Scientific Reports,* 13(1), 20075. 10.1038/s41598-023-47364-6 PMC10654384 37974023

[bib10] Gibson, J. J. (1954). A theory of pictorial perception. *Educational Technology Research and Development,* 2(1), 3–23.

[bib11a] Haber, R. N., & Levin, C. A. (2001). The independence of size perception and distance perception. *Perception & Psychophysics,* 63(7), 1140–1152. 10.3758/bf03194530 11766940

[bib11] Hart, S. G., & Staveland, L. E. (1988). Development of NASA-TLX (Task Load Index): Results of empirical and theoretical research. In P. Hancock, & N. Meshkati (Eds.), *Human mental workload* (pp. 139–183). Amsterdam: Elsevier.

[bib12] Interrante, V., Ries, B., & Anderson, L. (2006). Distance perception in immersive virtual environments, revisited. In *IEEE Virtual Reality Conference* (*VR* 2006) (pp. 3–10). Piscataway, NJ: Institute of Electrical and Electronics Engineers.

[bib13] Ittelson, W. H. (1951). Size as a cue to distance: Static localization. *The American Journal of Psychology,* 64(1), 54–67. 14819380

[bib14] Jitsumori, M. (2010). Do animals recognize pictures as representations of 3D objects? *Comparative Cognition & Behavior Reviews,* 5, 136–138.

[bib14a] Kim, N. G. (2018). Independence of size and distance in binocular vision. *Frontiers in Psychology,* 9, 988. 10.3389/fpsyg.2018.00988 PMC6026807 29988581

[bib15] Kim, J. J.-J., & Harris, L. R. (2022). Can people infer distance in a 2D scene using the visual size and position of an object? *Vision (Basel),* 6(2), 25. 10.3390/vision6020025 PMC9149917 35645379

[bib16] Kim, J. J.-J., & Harris, L. R. (2024). Updating the remembered position of targets following passive lateral translation. *PLoS One,* 19(12), e0316469. 10.1371/journal.pone.0316469 PMC11687815 39739643

[bib17] Kunnapas, T. (1968). Distance perception as a function of available visual cues. *Journal of Experimental Psychology,* 77(4), 523. 5672260

[bib18] Lappin, J. S., Shelton, A. L., & Rieser, J. J. (2006). Environmental context influences visually perceived distance. *Perception & Psychophysics,* 68(4), 571–581. 10.3758/bf03208759 16933422

[bib19] Leyrer, M., Linkenauger, S. A., Bülthoff, H. H., Kloos, U., & Mohler, B. (2011). The influence of eye height and avatars on egocentric distance estimates in immersive virtual environments. In *Proceedings of the ACM SIGGRAPH Symposium on Applied Perception in Graphics and Visualization* (pp. 67–74). New York: Association for Computing Machinery.

[bib20] Maltz, M. V., Stubbs, K. M., Quinlan, D. J., Rzepka, A. M., Martin, J. R., & Culham, J. C. (2021). Familiar size affects the perceived size and distance of real objects even with binocular vision. *Journal of Vision,* 21(10), 1–18, 10.1167/jov.21.10.21. PMC8479574 34581767

[bib21] Mark, L. S. (1987). Eyeheight-scaled information about affordances: A study of sitting and stair climbing. *Journal of Experimental Psychology: Human Perception and Performance,* 13(3), 361. 10.1037//0096-1523.13.3.361 2958585

[bib22] Mather, G., & Smith, D. R. (2000). Depth cue integration: Stereopsis and image blur. *Vision Research,* 40(25), 3501–3506. 10.1016/s0042-6989(00)00178-4 11115677

[bib23] Meehan, J. W., & Triggs, T. J. (1992). Apparent size and distance in an imaging display. *Human Factors: The Journal of the Human Factors and Ergonomics Society,* 34(3), 303–311. 10.1177/001872089203400305 1634242

[bib24] Messing, R., & Durgin, F. H. (2005). Distance perception and the visual horizon in head-mounted displays. *ACM Transactions on Applied Perception* (*TAP*), 2(3), 234–250.

[bib25a] Mon-Williams, M., & Tresilian, J. R. (1999). The size-distance paradox is a cognitive phenomenon. *Experimental Brain Research,* 126(4), 578–582. 10.1007/s002210050766 10422721

[bib25] Ooi, T. L., Wu, B., & He, Z. J. (2001). Distance determined by the angular declination below the horizon. *Nature,* 414(6860), 197–200. 10.1038/35102562 11700556

[bib26] Ozkan, K., & Braunstein, M. L. (2010). Background surface and horizon effects in the perception of relative size and distance. *Visual Cognition,* 18(2), 229–254. 10.1080/13506280802674101 PMC2929966 20814449

[bib27] Prabaswari, A. D., Basumerda, C., & Utomo, B. W. (2019). The mental workload analysis of staff in study program of private educational organization. *IOP Conference Series: Materials Science and Engineering,* 528(1), 012018.

[bib28] R Core Team. (2022). *R: A language and environment for statistical computing* (version 4.2.2). Vienna: R Foundation for Statistical Computing.

[bib29] Riecke, B. E., Behbahani, P. A., & Shaw, C. D. (2009). Display size does not affect egocentric distance perception of naturalistic stimuli. In *APGV ’09: Proceedings of the 6th Symposium on Applied Perception in Graphics and Visualization* (pp. 15–18). New York: Association for Computing Machinery.

[bib30] Rogers, S. (1996). The horizon-ratio relation as information for relative size in pictures. *Perception & Psychophysics,* 58(1), 142–152. 10.3758/bf03205483 8668514

[bib31] Roscoe, S. N. (1984). Judgments of size and distance with imaging displays. *Human Factors: The Journal of the Human Factors and Ergonomics Society,* 26(6), 617–629. 10.1177/001872088402600601 6532940

[bib32] Sedgwick, H. A. (2021). J. J. Gibson's “ground theory of space perception”. *i-Perception,* 12(3), 204166952110211. 10.1177/20416695211021111 PMC8334293 34377427

[bib33] Teghtsoonian, M., & Teghtsoonian, R. (1969). Scaling apparent distance in natural indoor settings. *Psychonomic Science,* 16(6), 281–283.

[bib34] Wickham, H. (2016). *Ggplot*2*: Elegant graphics for data analysis* (pp. 189–201). London: Springer Nature.

[bib35] Witt, J. K., Stefanucci, J. K., Riener, C. R., & Proffitt, D. R. (2007). Seeing beyond the target: Environmental context affects distance perception. *Perception,* 36(12), 1752–1768. 10.1068/p5617 18283926

[bib36] Wu, B., Ooi, T. L., & He, Z. J. (2004). Perceiving distance accurately by a directional process of integrating ground information. *Nature,* 428(6978), 73–77. 10.1038/nature02350 14999282

